# Improved Photoresponse of UV Photodetectors by the Incorporation of Plasmonic Nanoparticles on GaN Through the Resonant Coupling of Localized Surface Plasmon Resonance

**DOI:** 10.1007/s40820-020-00437-x

**Published:** 2020-04-13

**Authors:** Sundar Kunwar, Sanchaya Pandit, Jae-Hun Jeong, Jihoon Lee

**Affiliations:** Department of Electronic Engineering, College of Electronics and Information, Kwangwoon University, Nowon-gu, Seoul, 01897 South Korea

**Keywords:** UV photodetection, NP-based photodetectors, Nanoparticles, Plasmonic enhancement

## Abstract

Very small metallic nanostructures, i.e., plasmonic nanoparticles (NPs), can demonstrate the localized surface plasmon resonance (LSPR) effect, a characteristic of the strong light absorption, scattering and localized electromagnetic field via the collective oscillation of surface electrons upon on the excitation by the incident photons. The LSPR of plasmonic NPs can significantly improve the photoresponse of the photodetectors. In this work, significantly enhanced photoresponse of UV photodetectors is demonstrated by the incorporation of various plasmonic NPs in the detector architecture. Various size and elemental composition of monometallic Ag and Au NPs, as well as bimetallic alloy AgAu NPs, are fabricated on GaN (0001) by the solid-state dewetting approach. The photoresponse of various NPs are tailored based on the geometric and elemental evolution of NPs, resulting in the highly enhanced photoresponsivity of 112 A W^−1^, detectivity of 2.4 × 10^12^ Jones and external quantum efficiency of 3.6 × 10^4^% with the high Ag percentage of AgAu alloy NPs at a low bias of 0.1 V. The AgAu alloy NP detector also demonstrates a fast photoresponse with the relatively short rise and fall time of less than 160 and 630 ms, respectively. The improved photoresponse with the AgAu alloy NPs is correlated with the simultaneous effect of strong plasmon absorption and scattering, increased injection of hot electrons into the GaN conduction band and reduced barrier height at the alloy NPs/GaN interface.

**Electronic supplementary material:**

The online version of this article (10.1007/s40820-020-00437-x) contains supplementary material, which is available to authorized users.

## Highlights

- Enhancement of UV photoresponse by the incorporation of various plasmonic nanoparticles in the detector architecture.
- Detailed explanation for the photocurrent enhancement mechanism by the finite-difference time domain (FDTD) simulation and strong plasmon absorption.
- Systematic comparison and demonstration of the superior photoresponse of homogeneously alloyed AgAu nanoparticles as compared to the monometallic nanoparticles.

**Electronic supplementary material:**

The online version of this article (10.1007/s40820-020-00437-x) contains supplementary material, which is available to authorized users.

## Graphical abstract

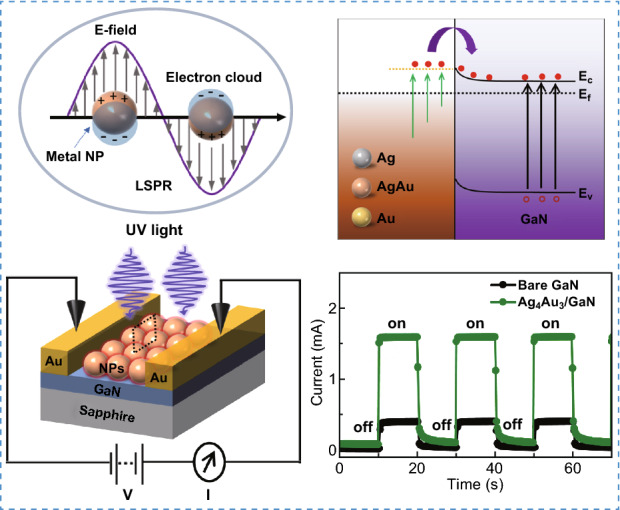

## Introduction

In recent years, ultraviolet (UV) detection has gained tremendous research attentions inspired by a wide range of applications in optoelectronics, environmental and biological monitoring, industrial automation, flame sensing, military communication, and space exploration [1–10]. The UV photodetectors feature the high sensitivity, fast response, low power consumption, and stable operation and thus can be utilized in the various aforementioned applications [11–13]. As an example, the UV communication system has been successfully developed for the unattended ground sensor networks, owing to the less interference from the visible light and insensitivity to the meteorological conditions and scatterings [10]. At the same time, the plasmonic nanomaterials that induce the localized surface plasmon resonances (LSPR) due to the collective oscillation of conduction band electrons have been gaining considerable research attention as the promising candidates to achieve the improved performance of various photodetectors [6, 14]. For example, the Cu nanostructure/ZnO quantum dots hybrid architecture exhibited the ultrahigh UV photoresponsivity due to the enhanced plasmon scattering by the Cu nanostructures in the ZnO photoactive layer [15]. To date, various UV photodetectors have been demonstrated by the modification of nanoscale surface properties of photoactive materials, i.e., wide bandgap semiconductors of GaN, ZnO, TiO_2_, and SiC, as well as by the integration of advanced nanomaterials such as metallic nanoparticles (NPs), two-dimensional materials, and quantum dots [16–20]. For instance, the fabrication of heterostructures nanowires of semiconductors such as bicrystalline GaN, Al-doped ZnO/ZnO nanorings/PVK/PE-DOT:PSS and crystalline silicon/porous silicon have been realized for high UV photoresponsivity and fast response speed [21–23]. In addition, the theoretical study of UV photodetector parameters such as active area, structure composition, and electrode materials could allow one to design a high-performance optoelectronic devices [24, 25]. Among various semiconductors, GaN has become a promising candidate for the UV detection due to its wide (~ 3.4 eV) direct bandgap, high carrier mobility, excellent chemical and thermal stability, and high electrical break down voltage [23]. In order to achieve the high responsivity, high quantum efficiency and faster response GaN-based UV photodetectors, the synthesis of nanostructured GaN layer, nanowires and integration of plasmonic metal NPs have been demonstrated [14, 17, 26]. However, the detailed study on the application of plasmonic AgAu alloy NPs in the UV photodetectors has not been attempted up to now. The Ag and Au NPs have shown excellent optical absorption, scattering, electromagnetic near-field, and charge transfer characteristics due to their strong LSPR effect in the UV–Vis regions [27–29]. Generally, the LSPR of Ag NPs is in the shorter wavelength (< 430 nm) as compared to the Au NPs (> 530 nm). Due to the synergistic effect of the Ag and Au, the AgAu alloy NPs can offer several advantages, i.e., a broader LSPR band, increased charge carrier density, longer carrier lifetime, and stability against oxide formation. On the other hand, the interfacial properties between plasmonic NPs and GaN such as Schottky barrier height can inherently be modified by the elemental composition of AgAu, which can be exploited to improve the excited carrier transfer between the plasmonic NPs and GaN. Thus, the composite alloy AgAu NPs can further boost the photoresponsivity, stability, and sensitivity of UV photodetectors for various practical optoelectronic devices.

**Fig. 1:**
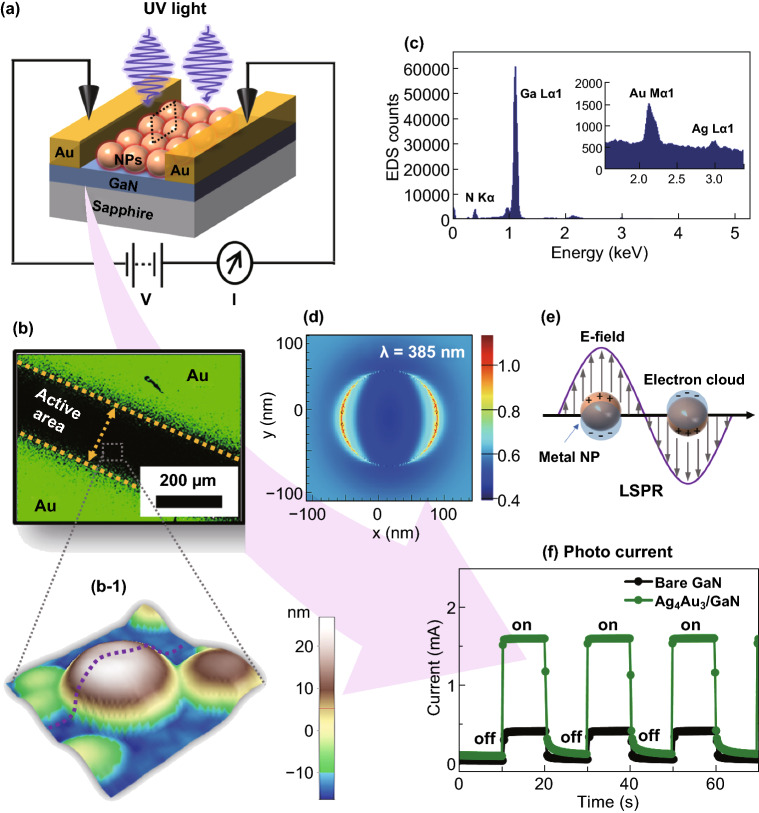
**a** Schematic of plasmonic metallic NPs based UV photodetector on GaN. **b** EDS elemental map of the photodetector active region and Au electrode. (**b-1**) AFM surface morphology of typical metallic NPs in the active region. **c** EDS spectra of NPs in the active region. **d** E-field distribution of typical metallic NP by finite-difference time domain (FDTD) simulation. **e** Schematic of localized surface plasmon resonance (LSPR) induced on the metal NPs by incident light. **f** Photoresponse of the bare GaN and Ag_4_Au_3_ at 0.1 V bias upon illumination of UV (385 nm) light

In this work, the significantly improved photoresponse of UV photodetectors by the incorporation of plasmonic NPs on GaN has been systematically demonstrated by the control of size and elemental composition as shown in Fig. 1. The maximum UV photoresponse is demonstrated with the higher Ag composition of AgAu alloy NPs, which exhibits a high responsivity of 112 A W^−1^, detectivity of 2.4 × 10^12^ Jones, and external quantum efficiency (*EQE*) of 3.6 × 10^4^% under UV illumination of 0.03 mW mm^−2^ at 0.1 V. The fully alloyed AgAu NPs and pure Ag and Au NPs are fabricated by the solid-state dewetting approach, which takes the advantages of atomic diffusion, interdiffusion and agglomeration upon annealing to form the definite shape and size of NPs. Depending upon the morphological and elemental evolution of the NPs, the photoresponse of corresponding device is dynamically varied due to the drastic variation in the LSPR response. Compared to the monometallic Ag and Au NPs, the alloy NPs exhibit high photoresponsivity, detectivity, and *EQE* due to the enhanced light absorption, scattering, hot electron excitation, and reduced barrier height at the GaN interface, which is correspondingly discussed along with the electromagnetic field simulation and energy band theory. Furthermore, the NPs based UV photodetectors also demonstrate an excellent photoresponse characteristic under zero bias (self-driven mode).

## Experimental Section

### Sample Preparation

In this study, various mono- and bimetallic NPs were fabricated on the epitaxially grown c-plane GaN (0001) template on sapphire (PAM-XIAMEN, China) and the samples were adapted for the fabrication of UV photodetectors. The GaN template was ~ 5 µm thick n-type with the resistivity < 0.5 ohms-cm and a dislocation density of < 1 × 10^8^ cm^−2^. Firstly, the large wafer was cleaved into 6 × 6 mm^2^ square pieces by a mechanical saw. Then, the substrates were subjected to a degassing process in a pulsed laser deposition (PLD) chamber at 600 °C for 30 min under 1 × 10^–4^ Torr to remove trapped oxides, particulates and water vapors. After degassing, the bare GaN surface morphology and optical characteristics were examined as shown in Fig. S1. The surface morphology clearly showed atomic step (< 0.6 nm average height) ripples of the GaN template.

### Fabrication of Nanoparticles and Photodetectors

Subsequently, various thickness of Ag, Au monolayer, and Ag/Au bilayer films were deposited on the clean GaN (0001) substrate. The deposition of metallic films was performed in a sputtering chamber under 1 × 10^–1^ torr at the deposition rate of 0.05 nm s^−1^ (20 s equals to 1 nm) and ionization current of 3 mA. First, the thin films of Ag (8 and 15 nm) and Au (3 and 5 nm) were deposited to fabricate pure Ag and Au NPs of different size on GaN. Second, two series of Ag_x_Au_y_ (x and y are the corresponding layer thickness) bilayers with a total thickness of 5 (Ag_3_Au_2_, Ag_2.5_Au_2.5_, Ag_2_Au_3_) and 7 nm (Ag_4_Au_3_, Ag_3.5_Au_3.5_, Ag_3_Au_4_) were deposited to fabricate AgAu alloy NPs with different size and elemental composition. For the fabrication of NPs, the Ag and AgAu bilayer films were annealed at 500 °C while the Au films were annealed at 650 ºC. For the fabrication of UV photodetectors, the Au electrodes were deposited on each NP sample and bare GaN as a reference as shown in Fig. 1a, b. A shadow mask of a 200-µm gap was placed on samples and then 100-nm-thick Au electrodes were deposited by sputtering. The photodetector devices were named according to the sample characteristics such as bare GaN, Ag, Au, and Ag_x_Au_y_.

### Characterizations and Simulation

The surface morphology of as-fabricated NPs was studied by a non-contact mode atomic force microscope (NC-AFM) (XE-70, Park Systems Corp. South Korea) and scanning electron microscope (SEM) (Regulus 8230, Hitachi, Japan). For the elemental characterizations of samples, energy-dispersive X-ray spectroscopes (EDS) (Noran System 7, Thermo Fisher, United States and Ultimax, Oxford Instruments, United Kingdom) were utilized. Optical characterization was performed by using a NOST system (Nostoptiks, South Korea), equipped with an ANDOR sr-500i spectrograph, CCD detector and combined deuterium-halogen light source (Ocean Optics, United Kingdom). Besides, the electromagnetic (EM) field and optical spectra of typical NPs on GaN were simulated by using a finite-difference time domain (FDTD) software (Lumerical Solutions, Canada). A plane wave source from 250 to 1100 nm in wavelength was used to excite the NPs on GaN from the *z*-direction. The perfectly matched layer (PML) boundary condition was applied in *z*-direction and the periodic boundary was applied in *x*, *y*-direction. The distance between the PML and the NP was half of the maximum wavelength to avoid interference. For the simulation, an auto-shut-off level of 10^−6^ and a 3D mesh grid of 0.5 nm were used. The complex refractive index of Au, Ag, and AgAu alloy was taken from the Rioux’s model [30]. Similarly, Kawashima’s model was referenced for the refractive index of GaN [31]. The photocurrent measurement of various devices was taken by using the B2902A precision source/measure unit (Keysight Technologies, USA). The high-power light-emitting diode (LED) of UV and VIS wavelengths (Mightex, Canada) was utilized to excite the photodetectors with the focus and collimator lenses with 10 mm focal length. The LED power was measured by a power meter (XLP12-3S-H2-D0, Genetec-eo, Canada). All the optical and photocurrent measurements were taken under dark and illumination at an ambient temperature.

## Results and Discussion

### Monometallic Ag/Au NPs and Growth Mechanism

**Fig. 2:**
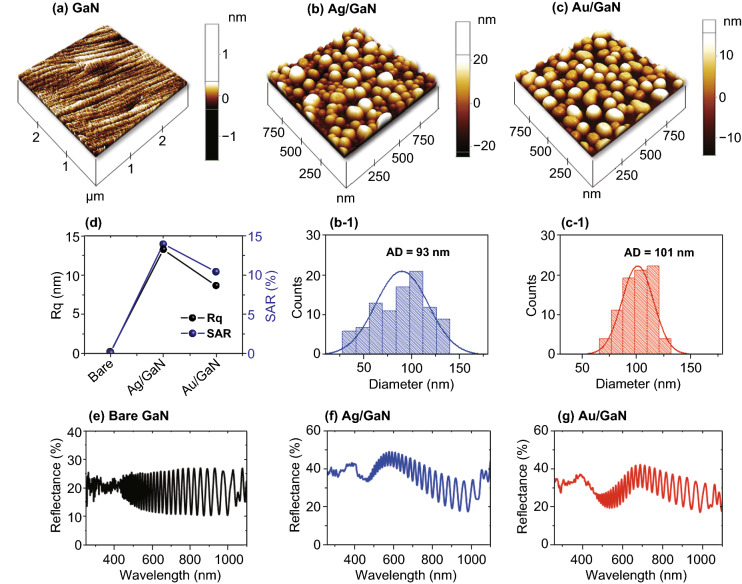
Morphological and optical analysis of bare GaN, Ag, and Au samples. **a**–**c** AFM side-views of the corresponding samples. **b-1**, **c-1** Diameter distribution histograms of the Ag and Au NPs on GaN. **d** Summary plots of Rq and SAR. **e**–**g** Reflectance spectra of bare GaN, Ag, and Au samples in the UV–VIS–NIR region

Figure 2 shows the morphological and optical properties of the bare GaN, monometallic Ag, and Au samples. The surface morphology of each sample is demonstrated by the 3D-AFM side-views and the large-scale AFM top-views are provided in Fig. S2. In addition, the dimensions of the Ag and Au NPs are studied by the RMS roughness (*R*_q_), surface area ratio (*SAR*), and average diameter distribution histograms. As shown in Fig. 2a, the surface of bare GaN showed the smooth atomic ripples on the surface after degassing. With the deposition of thin metallic films on GaN and subsequent annealing, the well-developed and isolated Ag and Au NPs were obtained owing to the solid-state dewetting well below the melting point of each metallic element [32, 33]. Specifically, the deposited metallic atoms can undergo sufficient thermal energy-driven surface diffusion and agglomerate in order to reduce the surface and interface energies [34]. The diffusivity (*D*_*s*_) of atoms directly depends upon the temperature of the system as below as Eq. 1 [35]:

1 $${D}_{s}\propto {e}^{-{E}_{a}/kT}$$

where the *E*_*a*_ is the activation energy of metal, *k* is the Boltzmann constant, and *T* is the annealing temperature. Besides, the evolution of NPs can be affected by the intrinsic properties of different metals, initial thickness, and annealing duration. Thus, the initial thickness and growth conditions for Ag and Au films were chosen to achieve the similar size and density of Ag and Au NPs. In specific, the initial thickness of Ag and Au was 15 and 5 nm for which the applied annealing conditions were 500 °C for 180 s and 650 °C for 450 s, respectively. After annealing at specified temperatures and duration under vacuum, the isolated and evenly distributed Ag and Au NPs were formed as displayed in Fig. 2b, c. The average diameter of Ag and Au NPs was found to be ~ 93 and 101 nm, respectively, as shown in Figs. 2b-1 and c-1. In addition, the *R*q and *SAR* were also extracted from the AFM images and summarized in Fig. 2d. Since the bare GaN was smooth and flat, the *R*_q_ and *SAR* were less than 2 nm and 0.009%, respectively. With the fabrication of semi-spherical Ag and Au NPs, the *R*_q_ increased to 13.23 and 8.23 nm while the *SAR* to 13.9 and 10.4%, respectively. Furthermore, the optical properties of bare GaN, Ag, and Au samples were studied by the reflectance spectra in the UV–VIS–NIR regions (250–1100 nm). The bare GaN exhibited a flatter reflectance spectrum with the characteristic reflectance ripples as shown in Fig. 2e with an average reflectance of 19%. The strong ripples in the reflectance can be attributed by the interference between the reflected photons from the air/GaN and GaN/Sapphire interfaces as shown in Fig. S1f. In the case of NP samples, the average reflectance was largely increased due to the high reflectivity of Ag and Au as witnessed in Fig. 2f, g [36]. Meanwhile, the reflectance spectra of the NPs samples revealed the wavelength dependency in the UV and VIS regions likely due to the LSPR effect of the Au and Ag NPs [37]. In specific, the reflectance dip for Ag NPs was at ~ 450 nm, which was shorter than that for the Au NPs at 540 nm. Generally, the reflectance dips and peaks of Au NPs were red-shifted as compared to the Ag NPs as the LSPR of Au NPs occur at longer wavelength [38]. The UV and VIS reflectance dips can be correlated with the excitation of dipolar and quadrupolar or higher-order resonance modes on the NPs by incident photons [39].

### UV Photoresponse of Monometallic Ag and Au NPs

**Fig. 3:**
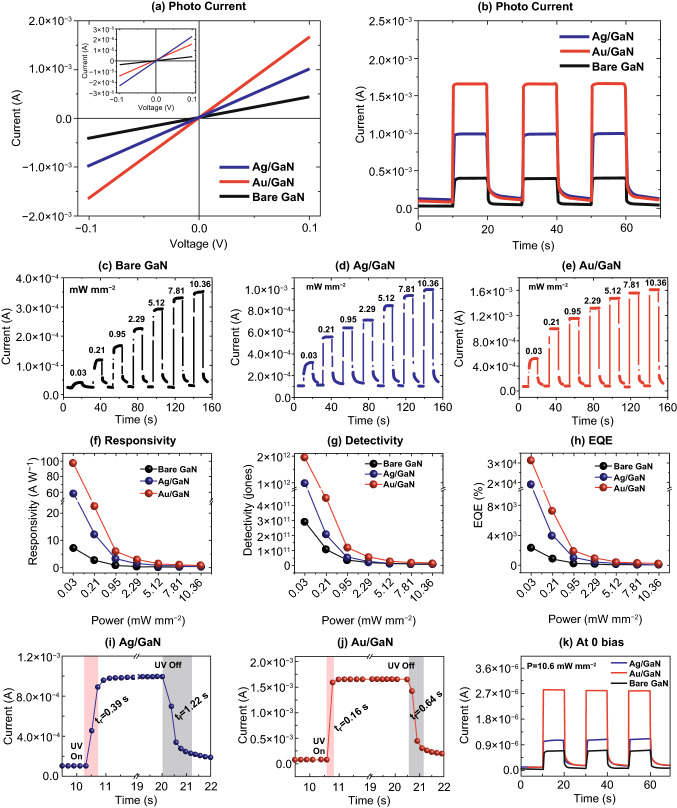
Photoresponse characteristics of the bare GaN, Ag and Au photodetectors. **a** Current–voltage (*I–V*) characteristics of each device under 385 nm UV illumination at 10.36 mW mm^−2^. Inset shows the corresponding dark current. **b** Current–time (*I–t*) characteristic at 0.1 V by switching on/off. **c**–**e** Photon power-dependent photoresponse of the devices. **f**–**h** Summary of the photoresponsivity (*R*), detectivity (*D*) and external quantum efficiency (*EQE*) as a function of photon power. **i**–**j** Rise (*t*_*r*_) and fall (*t*_*f*_) time of the Ag and Au detectors upon switching on/off. **k** Current–time (*I–t*) characteristic at 0 bias under 10.36 mW mm^−2^ UV illumination

Figure 3 presents the photoresponse of the bare GaN, Ag, and Au UV photodetectors excited by a power tunable UV-LED of 385 nm peak wavelength. A complete layout of the UV photodetectors is presented in Figs. 1a and S2a, describing the device architecture, active region, and light illumination scheme. For the measurement of linear current–voltage (*I–V*), the voltage range was varied between ± 0.1 V with the fixed power of 10.36 mW mm^−2^, illuminated on the active area of devices. The dark- and photocurrent of devices with and without NPs demonstrated significant differences as shown in Fig. 3a. Under dark, all three devices showed linearly increasing current in the order of 10^–6^ (bare GaN) to 10^–5^ A (NPs/GaN) at 0.1 V as shown in the inset of Fig. 3a, which indicates the quasi-Ohmic nature of devices [40]. It was observed that the dark current of Au and Ag devices was successively enhanced than the bare GaN, which could be mainly due to the thermionic emission and tunneling current [24]. At the same time, as the Schottky barrier height of Ag/GaN can be smaller than that of Au/GaN due to the lower work function, the dark current can increase further due to the increased thermionic emission of carriers [26]. The symmetrical characteristic of the *I–V* curve also indicates similar built-in electric field at the Au electrode/GaN junction and minimal surface state at the NPs/GaN interfaces [26, 41]. With the UV illumination, the photocurrent of bare, Ag and Au was sharply increased throughout the voltage range. For the detailed understanding of photoresponse behaviors, the pulsed UV light was illuminated at fixed 0.1 V as shown in Fig. 3b. Under an identical condition of applied voltage and photon power, the photocurrent of Ag and Au was found to be increased by ~ 2.8 and ~ 4.6 times, respectively, as compared to the bare as clearly seen in Fig. 3b. In specific, the photocurrent of bare GaN, Ag, and Au reached up to 3.56 × 10^–4^, 9.97 × 10^–4^, and 1.61 × 10^–3^ A, respectively, at 0.1 V and 10.36 mW mm^−2^. The integration of Ag and Au NPs on the bare GaN photodetector demonstrated a strong contribution toward photocurrent enhancement due to the LSPR effect [29, 42]. At the same time, it was also observed that the Au NPs induced much larger photocurrent as compared to the Ag NPs, which suggests the morphological and elemental dependence of LSPR in the photocurrent enhancement. Furthermore, the photon power-dependent photoresponse of each device was evaluated at fixed bias as shown in Fig. 3c–e. All three devices exhibited a consistent increment in the photocurrent with the power variation between 0.03 and 10.36 mW mm^−2^.

The device performance was further evaluated by determining the figure of merit parameters for photodetectors such as, responsivity (*R*), specific detectivity (*D*) and external quantum efficiency (*EQE*) at various power. The *R* is defined as the ratio of photocurrent generated to illuminated optical power intensity and expressed as Eq. 2 [29]:

2 $$R=\frac{{I}_{ph}-{I}_{d}}{{P}_{d}\times A}$$

where *I*_*ph*_is the photocurrent, *I*_*d*_ is the dark current, *P*_*d*_ is the power density (in mW mm^−2^) and *A* is the active area (in mm^2^) of device. According to the equation above the *R* was calculated at different optical power and plotted in Fig. 3f. In general, *R* was maximum at lower power, i.e., 0.03 mW mm^−2^ and was exponentially decreased at increased power, which could be attributed to the saturation of trap state in the GaN film for further injection of carriers [12]. The R was much enhanced with the incorporation of Ag and Au NPs. For example, the bare GaN showed 7.26 A W^−1^ while Ag and Au NP detectors showed 58.5 and 97.4 A W^−1^ at 0.03 mW mm^−2^. Furthermore, another parameter detectivity (*D*) denotes the photodetector sensitivity and calculated as Eq. 3:

3 $$D=R.{A}^{1/2}/{(2e{I}_{d})}^{1/2}$$

where, *R* is the calculated responsivity, *A* is the active area, *e* is the elementary charge and *I*_*d*_ is the dark current. From the calculation of *D*, it was also found to be significantly increased with the integration of Ag and Au NPs in the photodetectors as displayed in Fig. 3g. The Au photodetector exhibited a high value of 1.94 × 10^12^ Jones at the *P*_*d*_ of 0.03 mW mm^−2^, which is 1.98 and 6.67 times higher than Ag and bare GaN photodetectors respectively. In addition, the external quantum efficiency (*EQE*) that determines the ratio of photocurrent to the incident photon flux was determined as Eq. 4 [15]:

4 $$EQE=R \times \frac{1240}{\lambda} \times 100 \%$$

where *λ* is the excitation wavelength (385 nm). Similarly, the *EQE* of bare GaN, Ag, and Au photodetectors were maximum at 0.03 mW mm^−2^ and then gradually decreased with the increased power as shown in Fig. 3h. The *EQE* of Au detector was 3.13 × 10^4^, which was ~ 1.67 and 13.4 times higher than the Ag and bare GaN devices with the *EQE* of 1.88 × 10^4^ and 2.33 × 10^3^. The specific values of *R*, *D,*, and *EQE* are summarized at each power density in Table S2.

The response of the Ag and Au photodetectors was evaluated by the rise and fall time of photocurrent upon on–off switching as shown in Fig. 3i, j. Here, the rise time (*t*_*r*_) and fall time (*t*_*f*_) denote the time required to reach 90% of maximum photocurrent with the increase and the 10% of dark current while decreasing, respectively. The Au device demonstrated a shorter response time, i.e., *t*_*r*_: 0.16 s and *t*_*f*_: 0.64 s while the Ag detector showed 0.39 and 1.22 s, respectively. This can be due to the longer hot carrier lifetime of Ag NPs on the Fermi surface as compared with the Au NPs [43]. Figure 3k shows the photoresponse of devices under the illumination UV (10.36 mW mm^−2^) at zero bias. As all the devices fabricated in this work exhibited a non-zero dark current as shown in Fig. S2d likely due to the thermionic, thermionic-field emission, and minor built-in potential variation at the Au/GaN electrodes, they can be operated at zero bias [40]. A continuous three UV on–off cycles show the sharp rise and decay of photocurrent, suggesting the highly responsive self-driven UV photodetector operation [9, 23, 27]. The photoresponse of smaller Ag and Au NPs showed a similar trend with the relatively lower photocurrents as presented in Figs. S4 and S5.

### Characterization of Bimetallic AgAu Alloy NPs

**Fig. 4:**
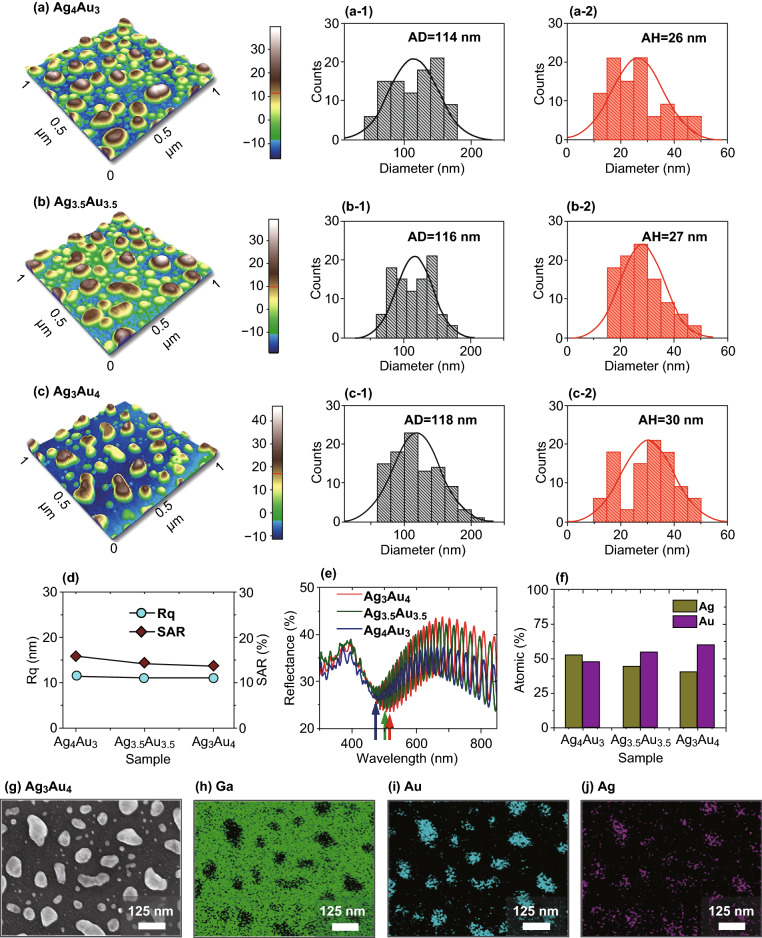
Surface morphology of AgAu alloy NPs fabricated on bare GaN with various thickness ratio of Ag and Au in the Ag_x nm_Au_y nm_ bilayers. **a**–**c** AFM images of the alloy NPs with the Ag_4_Au_3_, Ag_3.5_Au_3.5_ and Ag_3_Au_4_ bilayers on GaN_._ **a-1**–**c-1** Corresponding diameter distribution histograms of alloy NPs. **a-2**–**c-2** Height distribution histograms. **d** Summary plots of *R*_q_ and *SAR*. **e** Reflectance spectra. **f** Summary of the atomic percentage of Ag and Au with various thickness ratio. **g**–**j** SEM image and corresponding elemental maps of Ga, Au, and Ag for the alloy NPs fabricated with the Ag_3_Au_4_ bilayer

Figure 4 shows the fabrication of alloy NP photodetectors with the various Ag and Au compositions. To further explore the synergistic behavior of alloy NP UV photodetectors, the AgAu alloy NPs were fabricated with the Ag/Au bilayers on GaN. For the fabrication of AgAu alloy NPs, the total thickness of Ag/Au bilayer was 7 nm and the ratio of Ag and Au was controlled in order to realize the elemental variation in the alloy NP, i.e., Ag_4_Au_3_, Ag_3.5_Au_3.5_, and Ag_3_Au_4_ bilayers. As-deposited Ag/Au bilayers were annealed to induce sufficient surface diffusion, interdiffusion, and agglomeration of atoms, which finally yields the homogeneously intermixed alloy NPs below the melting point of metals [37]. As shown in Fig. 4a–c, the isolated and self-assembled AgAu alloy NPs of average diameter and height of ~ 116 and ~ 27 nm were obtained with the bilayers. Although the overall surface morphology of AgAu alloy NPs was similar, a minor deviation in size and configuration was observed due to the variation in the dewetting process with the different ratios of Ag and Au. It was also found that the diameter and height distribution was slightly wider than the pure Ag and Au NPs. In terms of the *R*_q_ and *SAR*, they were also found to be similar for all three types of AgAu alloy NPs as presented in Fig. 4d. In the case of reflectance spectra as shown in Fig. 4e, the alloy NPs clearly showed dips in the UV and VIS region corresponding to the quadrupolar or higher-order and dipolar resonance modes. Generally, the reflectance dips were between 480 and 516 nm, which were in the intermediate position between the pure Ag and Au NPs. With the increasing amount of Au in the alloy NPs, the VIS dips were gradually red-shifted as the LSPR of Au occur in the longer VIS wavelength. Meanwhile the average reflectance was decreased with the high Ag component, indicating the higher absorption in the UV–VIS region. Figure 4f summaries the atomic percentage of Ag and Au atoms in the alloy NPs. The fabricated AgAu alloy NPs were further investigated with SEM and elemental analysis of Ag_3_Au_4_ sample in Fig. 4g–j. As seen in SEM image and elemental phase maps, the NP sites clearly show the homogeneous distribution of Ag and Au atoms in NPs. The detailed morphological, elemental, and optical analyses are presented in Figs. S6–S9.

### Photoresponse of Bimetallic AgAu Alloy NPs

**Fig. 5:**
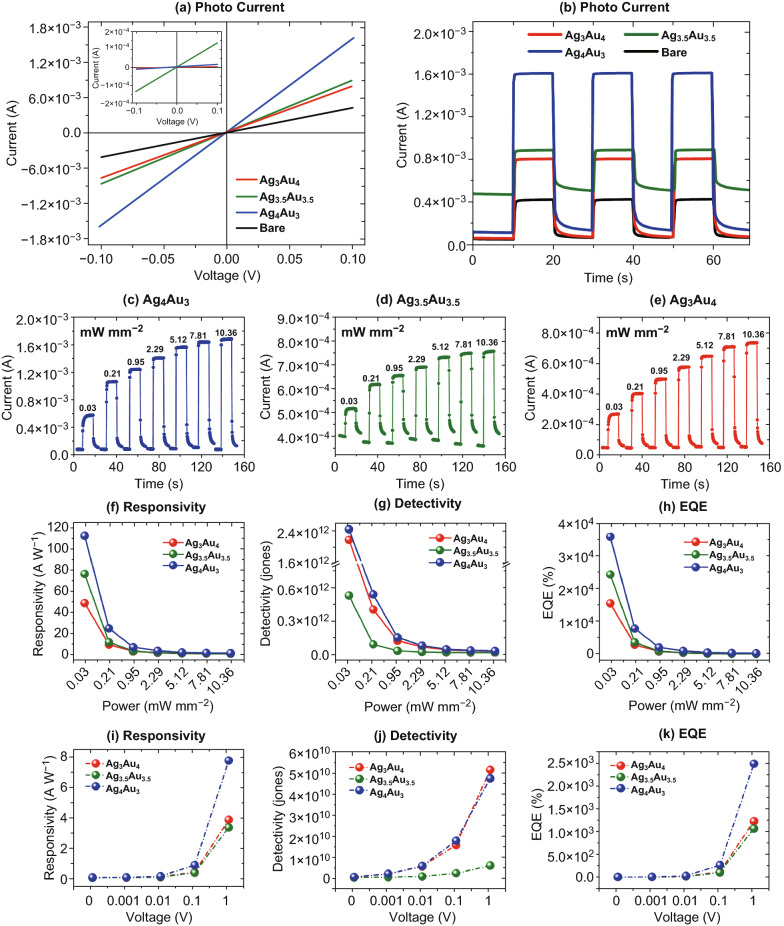
Photoresponse enhancement of UV photodetectors by the various AgAu alloy NPs under 385 nm UV illumination. **a** *I–V* characteristic under UV illumination and dark for different devices. **b** Time-resolved photocurrent response at 0.1 V. **c**–**e** Light intensity-dependent photoresponse of the devices. **f**–**h** Summary of the *R, D*, and *EQE* as a function of photon power at a constant bias of 0.1 V. **i**–**k** Summary of the *R, D*, and *EQE* as a function of voltage at a constant power of 10.36 mW mm^−2^

Figure 5 shows the photocurrent response of alloy NP UV detectors under the illumination of 385 nm at different power and bias voltages. Like the previous, the linear *I–V* response was observed at ± 0.1 V with the Ag_4_Au_3_, Ag_3.5_Au_3.5_, and Ag_3_Au_4_ detectors as shown in Fig. 5a. The dark current was higher with the AgAu alloy NPs. With the UV illumination, the Ag_4_Au_3_ device exhibited the highest photocurrent. The other Ag_3.5_Au_3.5_ and Ag_3_Au_4_ also showed much higher current as compared to the bare GaN as clearly seen in Fig. 5a, b. Depending upon the elemental composition of alloy NPs, the photocurrent of each device was drastically varied. It was also observed that the photocurrent sharply increased upon turning-on the UV and decay after turning-off for multiple on/off cycles, which infers fast response, good stability, and repeatability of the UV photodetectors. The power-dependent photocurrent enhancement was studied at a constant bias of 0.1 V as shown in Fig. 5c–e. Generally, all three devices showed an increasing trend with the power increase, in which the Ag_4_Au_3_ again showed more dynamic and highest photocurrent. The *R*, *D*, and *EQE* for the three photodetectors were calculated and is plotted in Fig. 5f–h. Compared to the pure Ag and Au NP devices, the Ag_4_Au_3_ alloy exhibited a higher photoresponse. For instance, the *R*, *D*, and *EQE* of Ag_4_Au_3_ were 112 A W^−1^, 2.4 × 10^12^ jones, and 3.6 × 10^4^%, respectively, at 0.03 mW mm^−2^ of illumination power, which were higher than those of previously reported GaN-based UV photodetectors with Ag NPs [26], with various GaN films [40, 44–48], and with graphene layers [49–51] as summarized in Table 1. However, the Ag_3.5_Au_3.5_ and Ag_3_Au_4_ devices exhibited somewhat moderate photoresponses with decreasing trend as the Au ratio was increased. Thus, the presence of both Ag and Au atom at specific alloy composition could result in the enhanced absorption of UV photons along with the photogenerated carrier in GaN. Specifically, the alloy NPs with the higher Ag percentage showed a better enhancement, which was contrary to the pure Ag and Au devices in the previous set and will be discussed in the later section. In terms of power-dependent photoresponse, all the alloy NP-based UV photodetectors showed a decreasing trend of the *R*, *D,* and *EQE* as clearly shown in summary plots in Fig. 5f–h. In addition, the performance of the UV photodetectors was examined under different biasing between 0 and 1 V, which showed high stability and repeatability as well as increased photocurrent response. Figure 5i–k summarizes the *R*, *D*, and *EQE* as a function of bias voltage at 10.36 mW mm^−2^, which clearly showed sharp increment in performance parameters with voltage due to the increased carrier drift velocity. The specific values of these device performance parameters are plotted and summarized in Fig. S10 and Tables S4 and S5.

**Table 1:** Comparision of performance parameters of the GaN-based UV photodetectors with various device configurations and materials reported in recent years

Materials	Light source (nm)	Bias (V)	*R* (A W^−1^)	*D* (jones)	*EQE* (%)	*T*_*r*_ (ms)	*T*_*f*_ (ms)
GaN nanoflowers [34]	325	1	10.5			67	24
Ag NPs/GaN [35]	360	5	4				
GaN film [40]	325	5	0.34	1.24 × 10^9^		280	450
Graphene/GaN [44]	325	10	0.361	2.3 × 10^10^	87.5	5.05	5.11
Graphene/GaN [45]	365	7	0.003				
r-GO/GaN [46]	350	0	0.0015	1.45 × 10^10^		60	267
β-Ga_2_O_3_/GaN [39]	365	5	3.7	4.7 × 10^10^	100		
a-plane GaN [41]	365	5	1.35			46	75
a-plane GaN [42]	365	2	0.74			57	13
ZnO/GaN [43]	300	0	0.176	2.5 × 10^12^		350	350
Ag_4_Au_3_/GaN	385	0.1	112	2.4 × 10^12^	3.6 × 10^4^	160	630

**Fig. 6:**
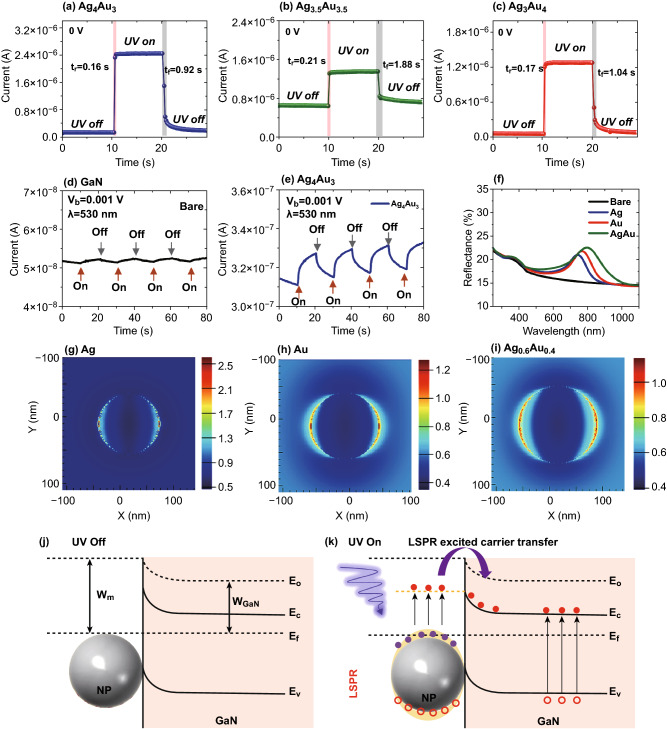
**a**–**c** Photoresponse of the Ag_4_Au_3_, Ag_3.5_Au_3.5_, and Ag_3_Au_4_ photodetectors under 385 nm UV illumination (10.36 mW mm^−2^) at 0 V. **d**, **e** *I–t* characteristic under the illumination of 530 nm at 1 mV for the bare GaN and Ag_4_Au_3_ photodetectors. **f** FDTD simulated reflectance spectra of the typical Ag, Au and Ag_0.6_Au_0.4_ alloy NPs. **g**–**i** Corresponding e-field distribution of the typical NPs at 385 nm excitation. **j**–**k** Schematics of the energy band diagrams of the NP photodetector and the charge transfer process under UV illumination

Figure 6a–c shows the self-driven characteristic of the UV photodetectors with the AgAu alloy NP detectors on GaN. The alloy NP photodetectors can be operated at zero bias with the decent stability and repeatability of photocurrent and the current was sharply increased at the UV illumination [32, 34, 36]. Comparing all three alloy NPs devices, the Ag_4_Au_3_ exhibited the highest photocurrent enhancement. Furthermore, it showed the shortest rise and fall time of 0.16 and 0.63 s, respectively, which is similar to the Au device. The fast response and self-driven characteristics of UV detectors are still highly demanded in the field of UV optoelectronics devices. Although much faster response speed and lower dark current of the GaN-based UV photodetectors have been reported recently with the application of 2D materials and various nanostructured surface morphology on GaN, the alloy NP-based UV photodetector demonstrated in this work was still superior in the overall performance as summarized in Table S5. For example, the *R*, *D*, and *EQE* are much higher with a decent rise and fall time of 0.16 and 0.63 s. In addition, the photoresponse of UV detector was further studied by the fabrication of smaller alloy NPs as presented in Figs. S11–S15 and Tables S6 and S7. Overall photodetector performance was slightly reduced as compared to the larger size alloy NPs, which can be correlated with the reduced plasmonic effect of smaller NP size. At the same time, the higher Ag concentration NP detector, i.e., Ag_3_Au_2_, demonstrated the highest photocurrent as well as the highest *R, D*, and *EQE* as a function of photon power variation. In addition, the photodetectors were evaluated in the VIS wavelength. Under the VIS illumination (530 nm at ~ 3 mW mm^−2^), the bare GaN photodetector exhibited almost negligible photocurrent as shown in Fig. 6d because of the photon energy less than the bandgap of GaN. At the same time, the photoresponse became noticeable and got stronger with the Ag_4_Au_3_ NPs, which can be correlated to the enhanced absorption of VIS wavelength due to the LSPR on the alloy NPs [27–29]. In contrast to the UV response, the VIS photoresponse was very minor and gradually increased.

### Photoresponse Enhancement Mechanism

Finally, the underlying mechanism associated with the photocurrent enhancement of the UV photodetectors with plasmonic NPs can be discussed based on the interaction between incident photon and NPs and energy band theory as shown in Fig. 6f–k. Firstly, the electromagnetic interaction of various NPs with incident photons was elucidated by the FDTD simulations. The simulated reflectance in Fig. 6f and localized e-fields of the typical Ag, Au, and Ag_4_Au_3_ NPs are presented in Fig. 6g–j and the transmittance and extinction spectra are provided in Fig. S8. The simulated spectra also clearly showed dips and peaks in the UV and VIS regions followed by peaks due to the LSPR of the NPs whereas nearly flat response was obtained with bare GaN [39]. The plasmonic effect was successively enhanced with the Ag, Au, and Ag_4_Au_3_ alloy NPs, which was indicated by the stronger peaks and dips in reflectance spectra. In addition, the localized e-field distribution of Ag, Au, and Ag_0.6_Au_0.4_ alloy NPs also exhibited much stronger and broader optical near-field enhancement accordingly. As seen in Fig. 6g–j, the e-field distribution of Ag NPs was mostly confined at the edges whereas the Au and Ag_0.6_Au_0.4_ alloy NPs showed much wider distribution. From the simulation of optical spectra and e-field, it was clear that the LSPR inherently varied based upon the surface morphology and the elemental composition of NPs such as the Ag_0.6_Au_0.4_ alloy NPs have the strongest LSPR and then gradually reduced with the Au and Ag NPs. On the other hand, upon UV excitation the Ag and Au NPs induce the intraband and interband excitation of electrons known as hot carriers associated with the nonradiative plasmonic decay [27, 52]. The excited surface plasmon in metal NPs can decay by generating and elelctron-hole pair with much larger energy than the carriers near the Fermi energy [53]. From the time-resolved photoresponse, it was found that the bare GaN and NPs/GaN samples show instant current increase after LED is switched on and become steady. This process implies that the response can be mainly dominated by the ultrafast hot electron injection rather than photothermal effect and localize e-field enhancement [52]. Although the photoresponce in our device could be partially contributed each of these effects but the hot electron excitation and injection can be more dominant phenomenon. It also should be noted that the hot electron injection efficiency vary with the composition of alloy NPs such that the higher Ag concentration can offer more hot electron transfer due to the shifting of interband threshold energy [27]. Therefore, as compared to the pure Au and Ag NPs, much-enhanced light absorption and hot electron transfer can result in the increased photocurrent generation with the alloy NP. Meanwhile, for the large size NPs, the LSPR effect can also be contributed by high forward scattering towards the GaN interface, which is more stronger with the Au than Ag and likely generates additional electron–hole pairs in GaN bandgap [54, 55]. In addition, the photocurrent enhancement of the UV photodetectors largely depends upon the energy band of metallic NPs and GaN. Generally, the work function of Ag (4.26–4.7 eV) and Au (5.10–5.47 eV) is higher than the n-type GaN (4.2 eV) and thus the energy band aligns as presented in Fig. 6j to match the Fermi level when the metal NPs and GaN are brought in contact under dark conditions [56]. However, the Schottky barrier height can vary for Ag, Au, and AgAu devices. For the complete miscible binary metal, the work function for the alloy composition can be estimated based on the mass fraction as given by the relation as Eq. 5:

5 $$\boldsymbol{\varnothing }\left({Ag}_{x}{Au}_{1-x}\right)=x \times \varnothing \left(Ag\right)+(1-x) \times \varnothing (Au)$$

where *Ø*(*Ag*) and *Ø*(*Au*) are the work functions of pure Ag and Au, respectively [57, 58]. Based on the work function of different metals, the Ag, AgAu, and Au could possess the lowest, medium, and highest barrier height, respectively. Therefore, the conductivity of Ag photodetector under dark conditions can be higher as compared to the pure Au. However, the Ag_3.5_Au_3.5_ showed exceptionally high dark current, which can be due to the difference in the surface composition and crystal facets that could also largely affect the work function in the alloy phase [59]. Under the UV light illumination, the energy from the photons can be absorbed by the GaN to generate electron–hole pairs as shown in Fig. 6j, which contributes to the photocurrent generation in the device. Meanwhile, the electromagnetic fields on the NPs can also be enhanced due to the collective oscillation of electrons and the excited hot electrons from the metallic NPs spontaneously transfer to the conduction band of GaN. Therefore, the overall photocurrent of the device can be largely enhanced by the plasmonic NPs on the GaN. In our case, the AgAu alloy composition with the higher Ag percentage exhibited the highest performance parameters as compared to other pure and alloy NPs, which could be attributed to the combined effect of enhanced plasmonic absorption and the decreased barrier height at the GaN interface.

## Conclusions

In summary, the photoresponse of UV photodetectors was studied with the mono- and bimetallic NPs of the different size and elemental compositions on GaN (0001) based on the solid-state dewetting approach. The dynamic and improved photoresponse of UV photodetectors has been demonstrated with various monometallic Ag and Au and bimetallic AgAu alloy NPs. Specifically, the AgAu alloy NPs with the higher Ag percentage exhibited the highest responsivity of 112 A W^−1^, detectivity of 2.4 × 10^12^ jones, and *EQE* of 3.6 × 10^4^% under 0.03 mW mm^−2^ at 0.1 V, which is a superior result as compared to the previously reported GaN-based UV photodetectors. The fabricated UV photodetectors were operated at comparatively low voltage, i.e., below 1 V, or even at no bias (self-driven mode) and have demonstrated very sensitive and stable photocurrent responses. In the alloy NPs, the increased concentration of Ag resulted in an improved performance as being benefitting from the enhanced light absorption and scattering, hot electron transfer and reduced barrier height at the GaN interface. Furthermore, the photocurrent was significantly enhanced without sacrificing the response time as compared to the bare GaN devices. The mechanism of photocurrent enhancement was systematically discussed with the help of FDTD simulation and band theory of metallic NPs and GaN. This work could be of great potentials for advancing GaN-based UV detectors.

## Electronic supplementary material

Below is the link to the electronic supplementary material.

Supplementary file1 (PDF 2739 kb)
