# Supplementary material for: Improved Photoresponse of UV Photodetectors by the Incorporation of Plasmonic Nanoparticles on GaN Through the Resonant Coupling of Localized Surface Plasmon Resonance
Source: Nanomicro Lett. 2020 Apr 13;12:91. doi: 10.1007/s40820-020-00437-x (PMC7770873; doi:10.1007/s40820-020-00437-x)
Supplement: Supplementary file 1 — Supplementary file1 (PDF 2739 kb) [file 40820_2020_437_MOESM1_ESM.pdf]

Supporting Information for

# Improved Photoresponse of UV Photodetectors by the Incorporation of Plasmonic Nanoparticles on GaN through the Resonant Coupling of Localized Surface Plasmon Resonance

Sundar Kunwar[1], Sanchaya Pandit[1], Jae-Hun Jeong[1], Jihoon Lee [1,] *

[1]Department of Electronic Engineering, College of Electronics and Information, Kwangwoon University, Nowon-gu Seoul 01897, South Korea

*Corresponding author. E-mail: jihoonlee@kw.ac.kr (Jihoon Lee)

## Supplementary Figures and Tables

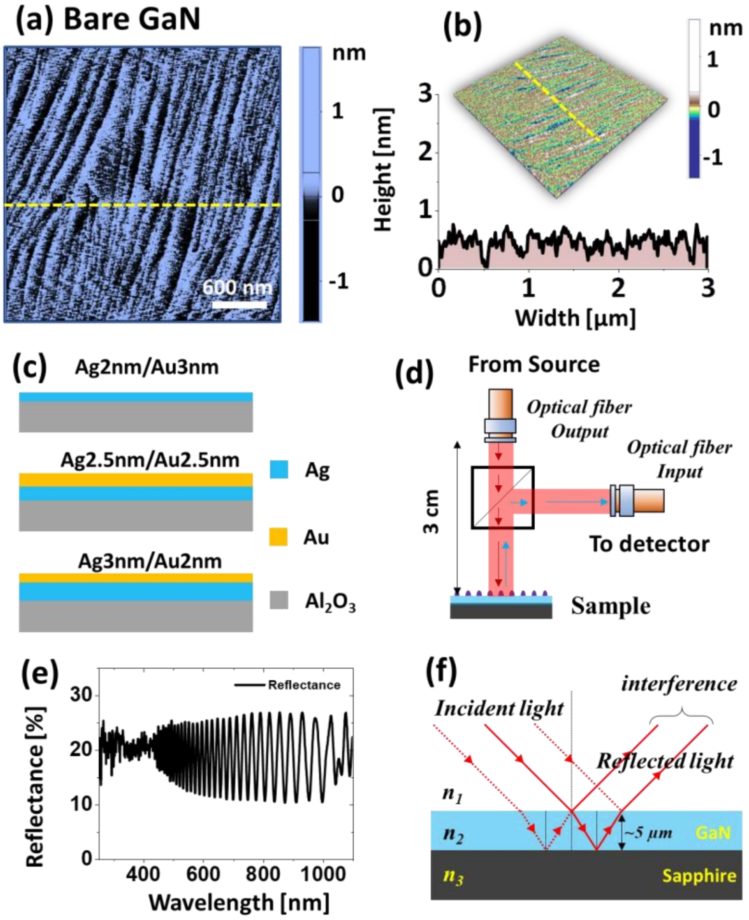


**Fig. S1 a** AFM images of bare GaN prior to the deposition of metallic films. **b** Corresponding AFM side-view and cross-sectional line-profile. **c** Schematic for the deposition of Ag, Au and Ag/Au bilayers for the fabrication of plasmonic NPs/GaN based UV photodetectors. **d** Schematic of UV-VIS-NIR reflectance measurement. **e** Reflectance spectra. **f** Schematic of interreference between reflected light between air/GaN and GaN/Sapphire interface

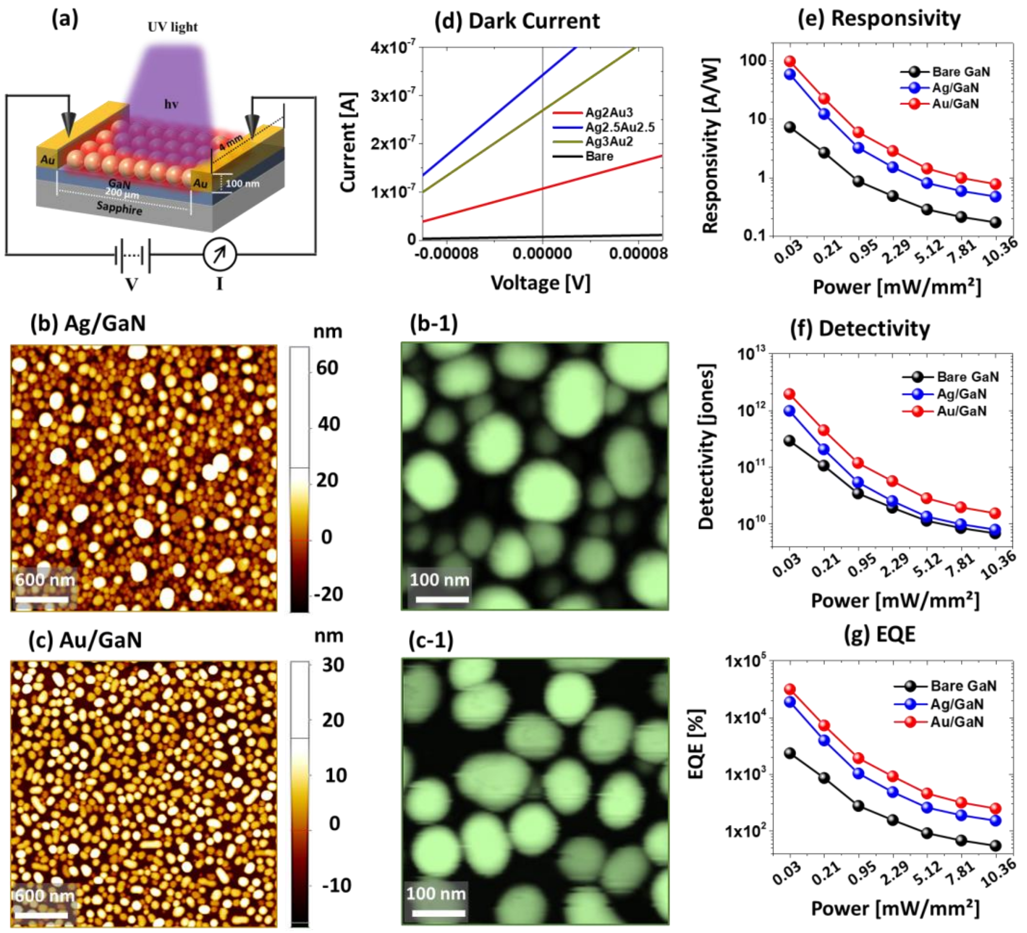


**Fig. S2 a** Schematic of the plasmonic NPs/GaN UV photodetector. The active region between two electrodes was 200 µm and the channel length was 4 mm and the sputtered gold electrodes were about 100 nm thick. **b**-**c** AFM top-views of the mono-metallic Ag and Au NPs fabricated on GaN. (**b-1**)-(**c-1**) Enlarged AFM images of the NPs. **d** Dark current of photodetector devices at low voltage range. **e-g** Summary plots of the responsivity (R), detectivity **d** and EQE of the bare GaN, Ag/GaN and Au/GaN photodetectors based on the light power variation (0.03 to 10.36 mW/mm$^2$). The excitation wavelength and bias voltage were 385 nm and 0.1 V respectively

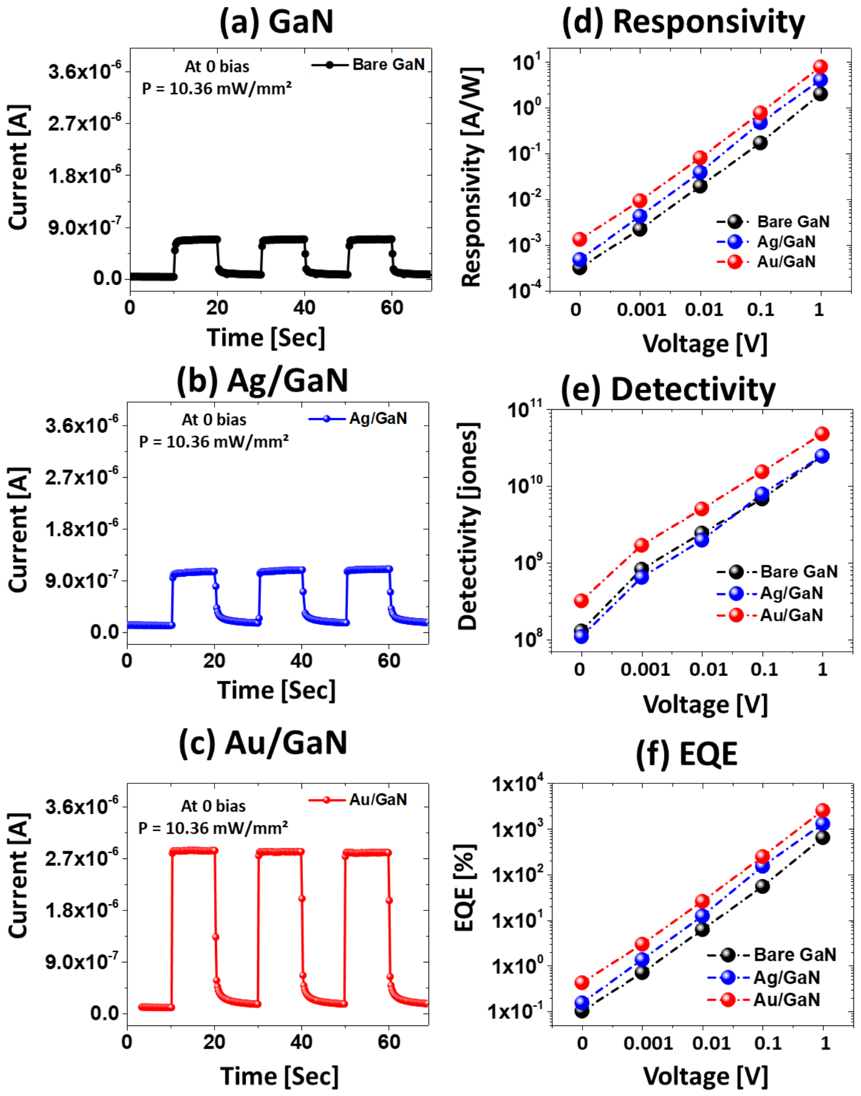


**Fig. S3 a-c** Photocurrent switching characteristics of the bare GaN, Ag and Au photodetectors at 0 bias upon UV on/off. The light intensity was fixed at 10.36 mW/mm$^2$. **d-f** Summary of the R, D and EQE of the photodetectors based on the voltage variation at constant light power of 10.36 mW/mm$^2$

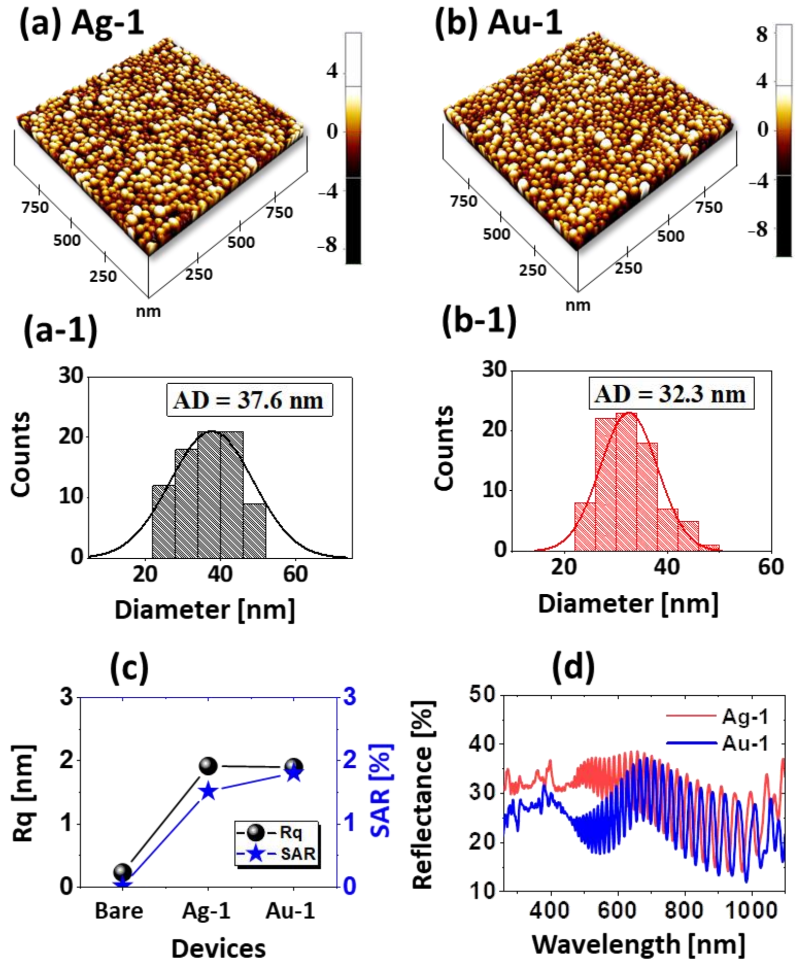


**Fig S4** Fabrication of smaller size Ag and Au NPs GaN. **a-b** AFM side-views. (**a-1**)-(**b-1**) Diameter distribution histograms. **c** Summary of the RMS roughness ($R_q$) and surface area ratio ($SAR$). The Rq provides an average of surface profile height ($y_i$) as**:** $R_q = \sqrt{\frac{1}{n}\sum_{i=1}^{n} y_i^2}$ . Whereas the SAR probes the increment of the 3D surface area of NPs ($A_{NP}$) with respect to the 2D geometric area ($A_s$) as: $SAR = \frac{A_s - A_{NP}}{A_s} \times 100\ \%$. **d** Reflectance spectra of Ag-1/GaN and Au-1/GaN

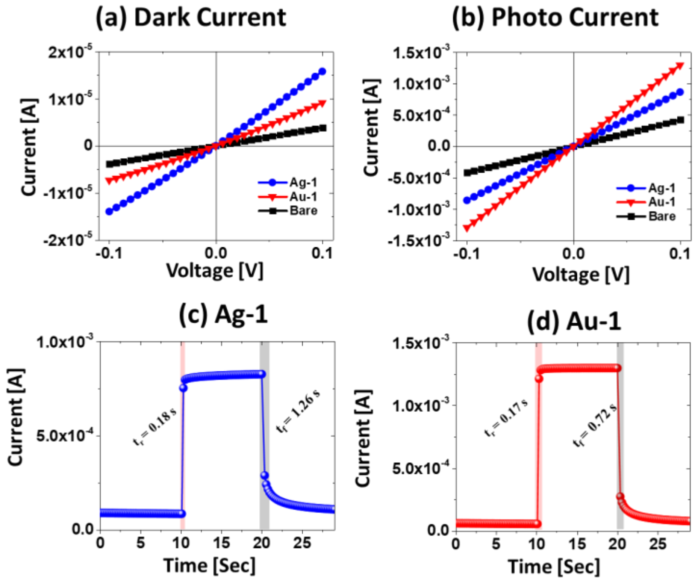


**Fig. S5** Photoresponse of the small size Ag-1/GaN and Au-1/GaN NPs photodetectors. **a** *I-V* characteristic under dark. **b** Corresponding photocurrent of the devices upon illumination of UV (385 nm) at 10.36 mW mm$^{-2}$. **c-d** *I-t* characteristics of Ag-1/GaN and Au-1/GaN photodetectors

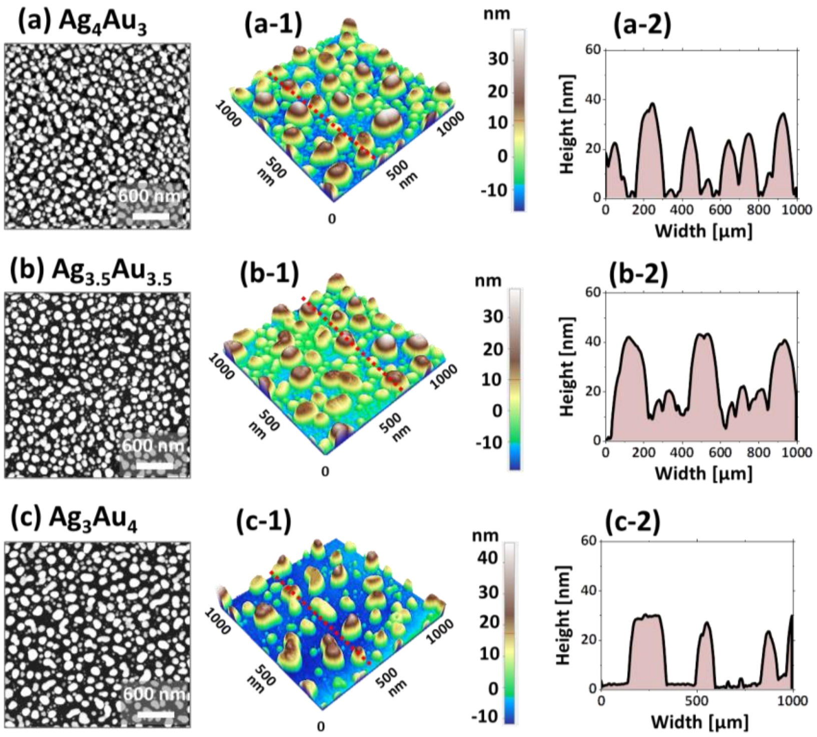


**Fig. S6 a-c** AFM images of the large AgAu alloy NPs fabricated on GaN with various thickness of Ag and Au in the bilayer configuration ($Ag_{x\ nm}Au_{y\ nm}$) as labelled. The AgAu alloy NPs were fabricated at 500 °C for 60 sec. (**a-1**)-(**c-1**) Corresponding AFM side-views. (**a-2**)-(**c-2**) Cross-sectional line profiles of the AFM images in **a-c**

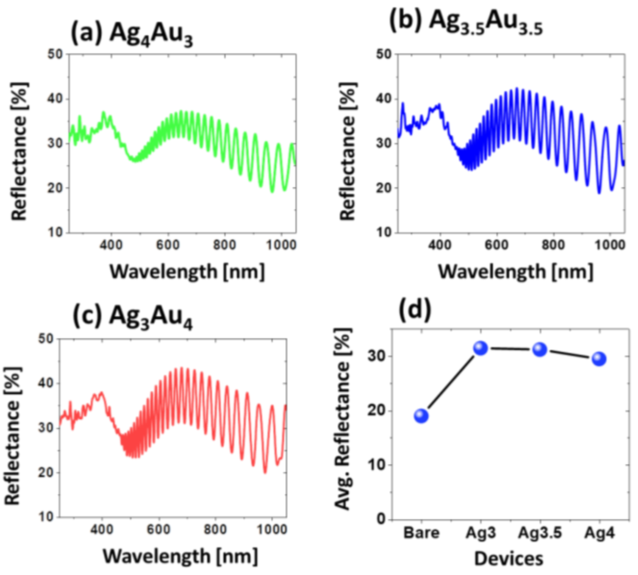


**Fig. S7 a-c** UV-VIS-NIR reflectance spectra of the large AgAu NPs on GaN fabricated with various initial thickness of Ag and Au. **d** Summary of the average reflectance for each sample

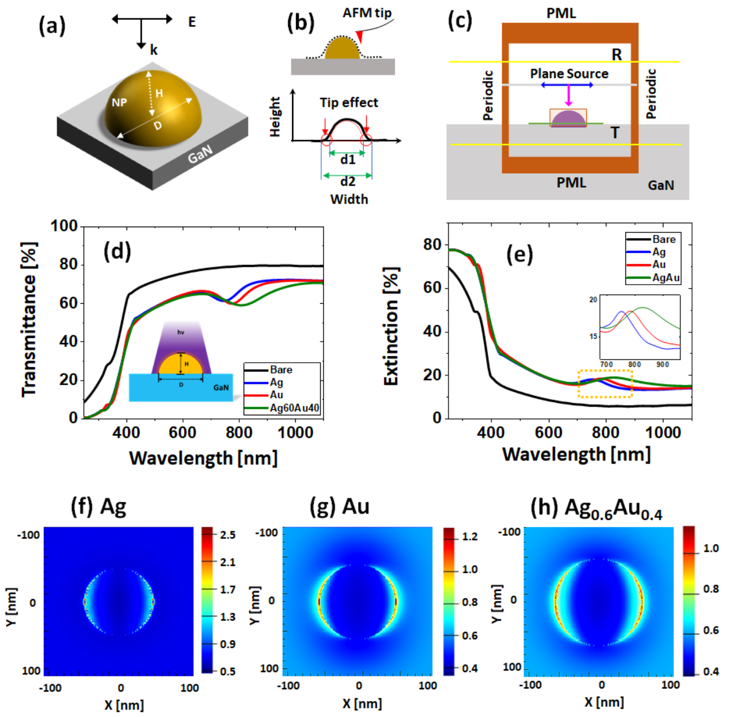


**Fig. S8 a** Three-dimensional model of semi-spheroid NP on GaN. **b** Diameter variation induced by the AFM tip effect. The d1 and d2 are the real and measure diameters. **c** Schematic of the finite difference time domain (FDTD) model used for the simulation. **d-e** FDTD simulated transmittance and extinction spectra of the Ag, Au and AgAu alloy NPs. **f-h** Corresponding e-field distribution of the Ag, Au and AgAu alloy NPs at 385 nm excitation

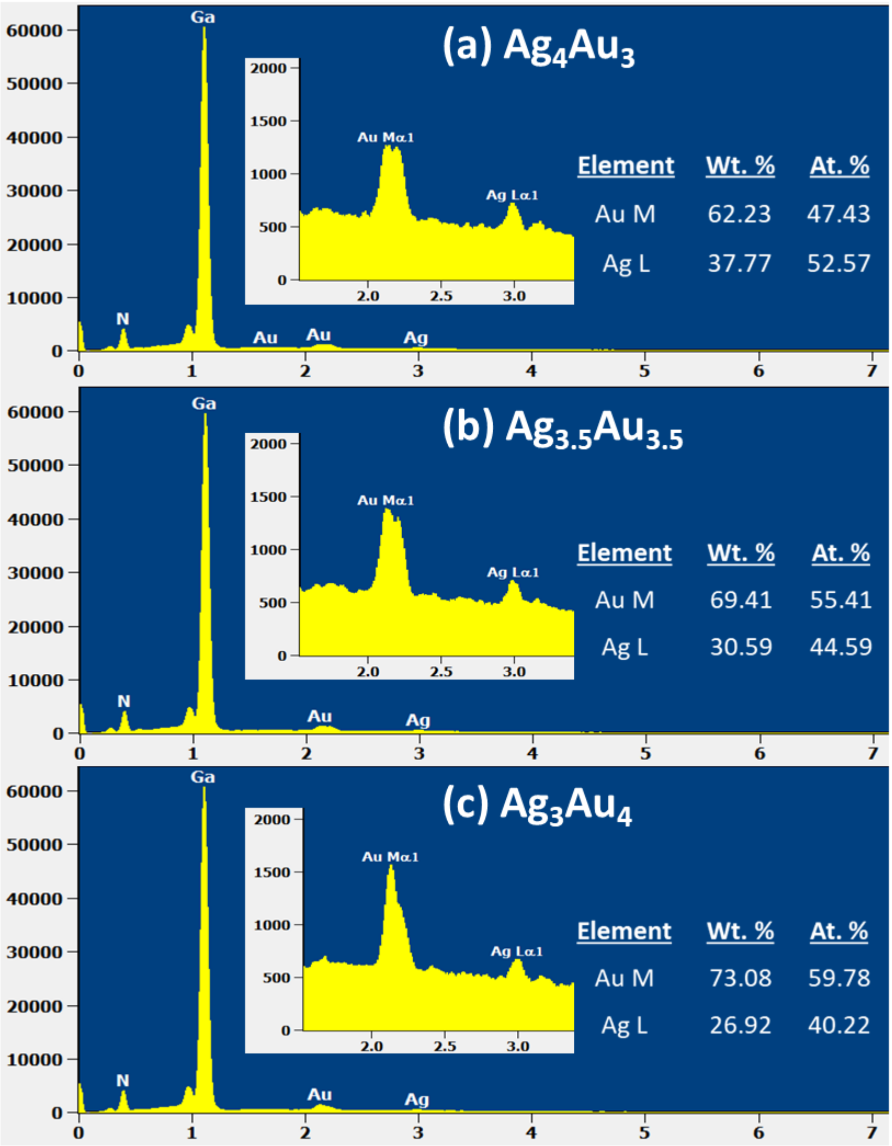


**Fig. S9 a-c** Energy dispersive x-ray spectroscope (EDS) spectra of the large size AgAu alloy on GaN fabricated with the various bilayer thickness of Ag and Au as labelled. The weight percentage (wt %) and atomic percentage (at%) are shown in inset tables

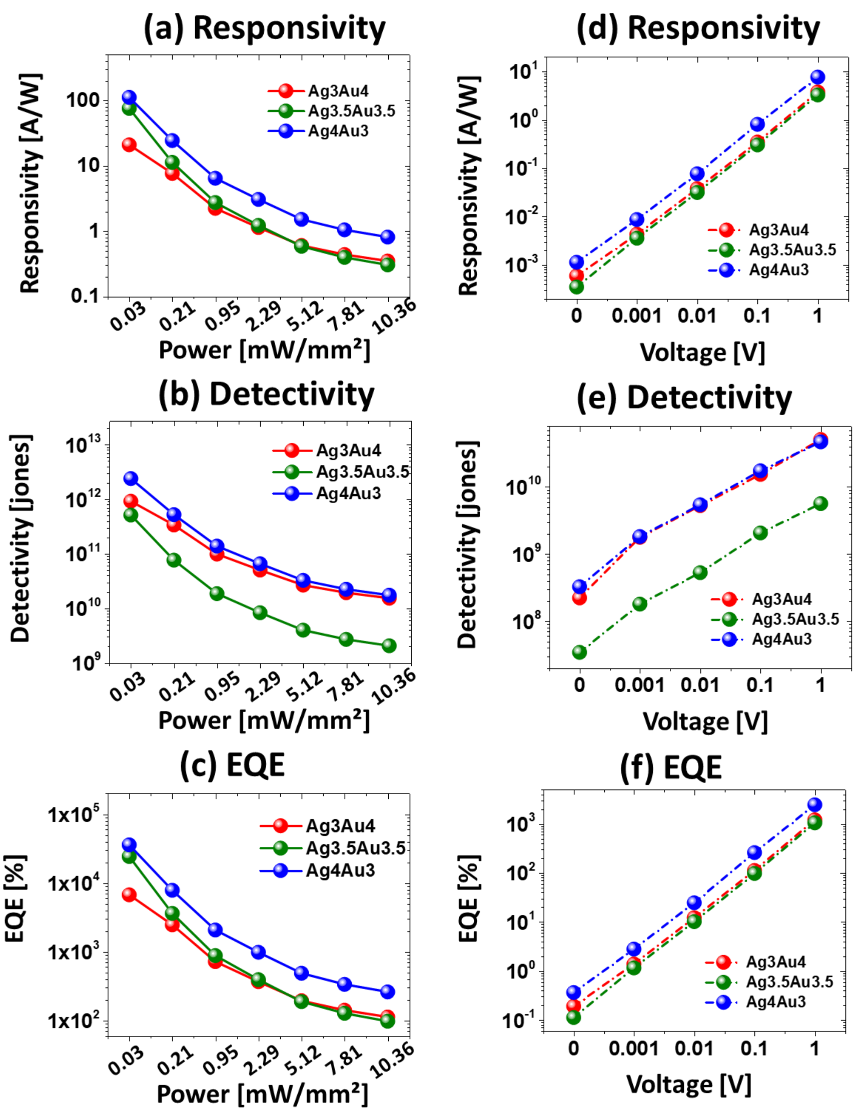


**Fig. S10 a-c** Summary plots of the R, D and EQE of the $Ag_4Au_3$/GaN, $Ag_{3.5}Au_{3.5}$/GaN and $Ag_3Au_4$/GaN photodetectors based on the photon power variation at fixed 0.1 V bias. **d-f** Summary of the *R*, *D* and *EQE* based on the voltage variation at fixed illumination of 385 nm at 10.36 mW $mm^{-2}$

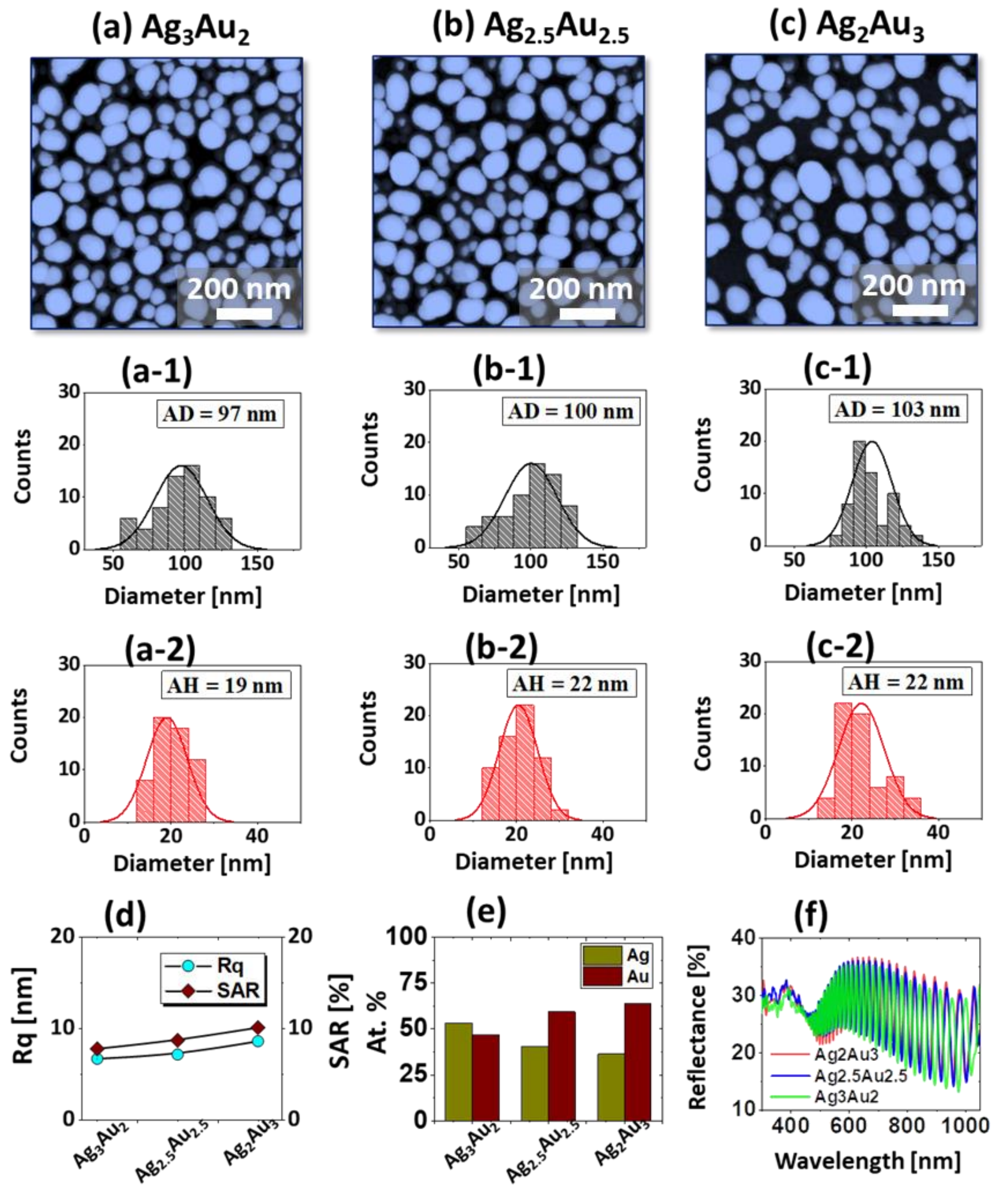


**Fig. S11** Fabrication of small AgAu alloy NPs on GaN with various $Ag_{x\ nm}Au_{y\ nm}$ bilayer composition with total thickness of 5 nm by annealing at 500 ºC for 60 sec. **a** – **c** AFM top-views of the small AgAu alloy NPs. (**a-1**) – (**c-1**) Diameter distribution histogram. (**a-2**) – (**c-2**) Height distribution histogram. **d** Summary of the Rq and SAR. **e** Summary of At. % of Ag and Au in alloy NPs. **f** Reflectance spectra of the small alloy NPs

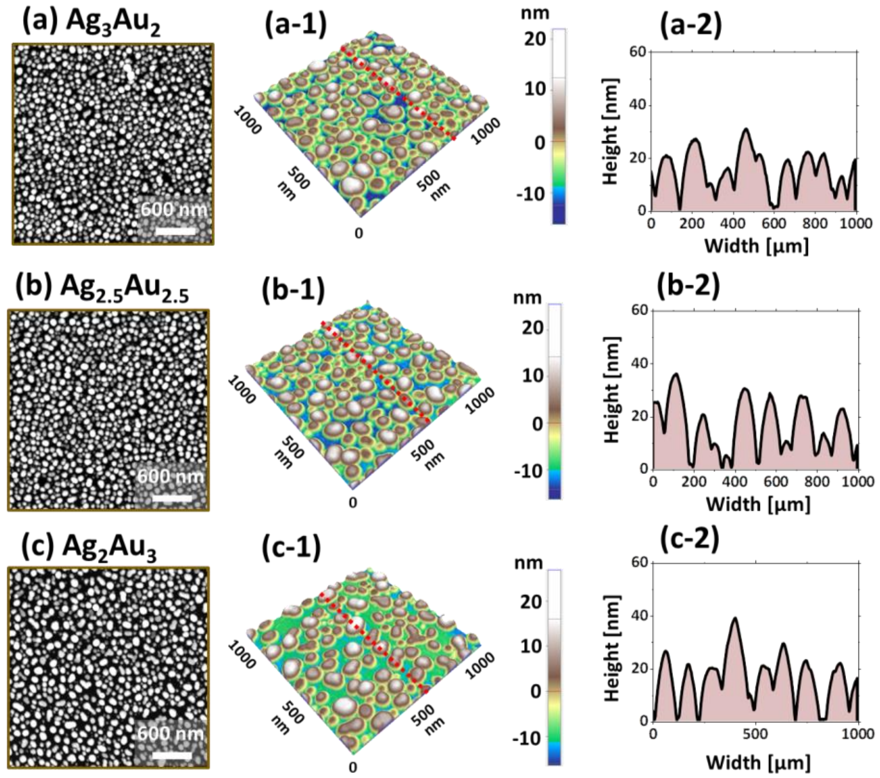


**Fig. S12 a-c** Large scale AFM images of the small AgAu NPs fabricated on GaN with various thickness of Ag and Au in the bilayer ($Ag_{x\ nm}Au_{y\ nm}$) as labelled. The AgAu alloy NPs were fabricated at 500 ºC for 60 sec. (**a-1**) - (**c-1**) Corresponding AFM side-views. (**a-2**) – (**c-2**) Cross-sectional line profiles of the AFM images in **a-c**

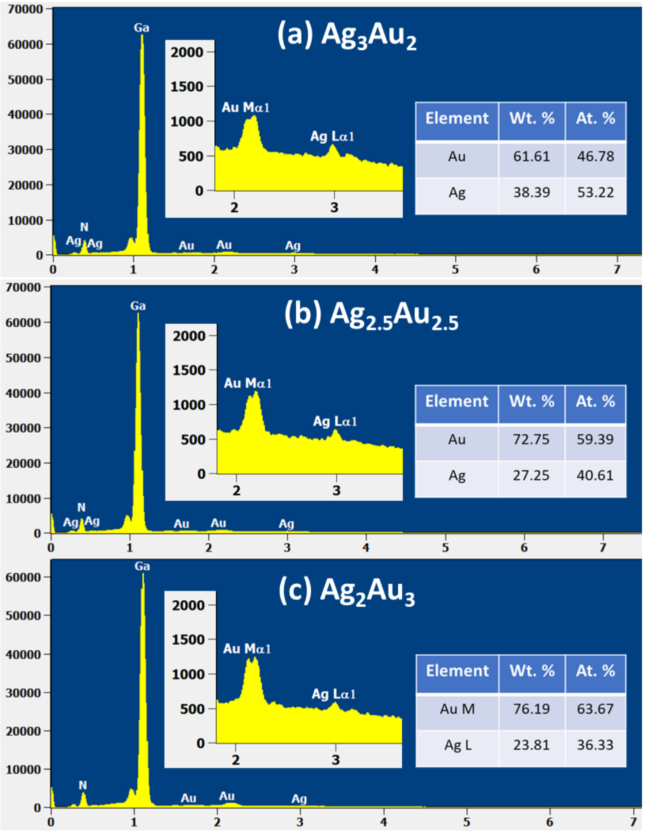


**Fig. S13 a-c** EDS spectra of the small size AgAu alloy on GaN fabricated with the various bilayer thickness of Ag and Au as labelled. The wt% and at% are shown in inset tables

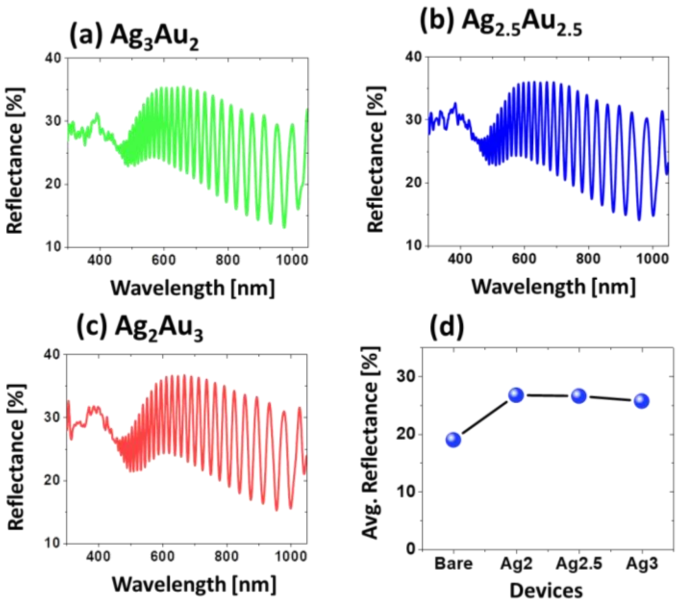


**Fig. S14 a-c** UV-VIS-NIR reflectance of the small AgAu NPs on GaN fabricated with various initial thickness of Ag and Au. **d** Summary of the average reflectance for various samples

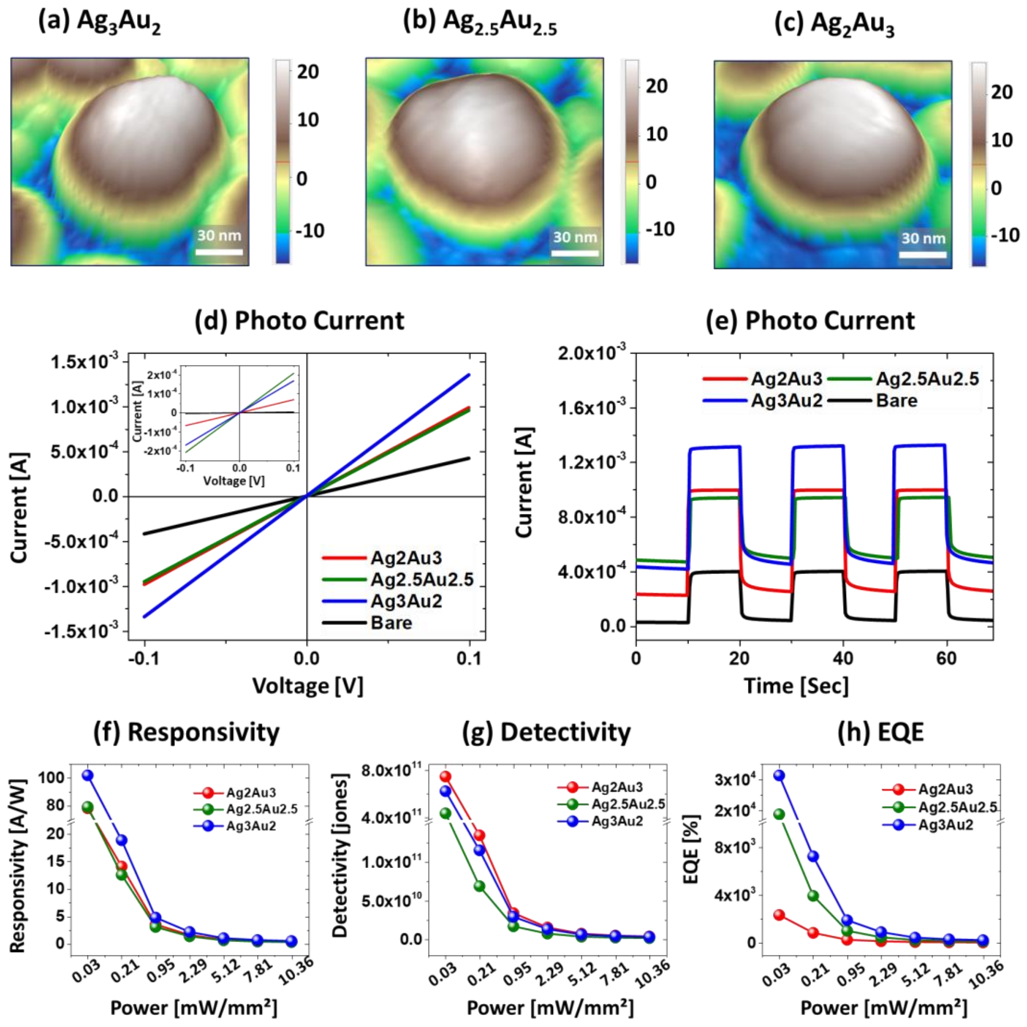


**Fig. 15 a-c** Typical AFM images of the small AgAu alloy NPs fabricated with various $Ag_xAu_y$ thickness ratio on GaN as labelled. **d** *I-V* characteristic of the smaller AgAu/GaN photodetectors under 385 nm illumination at 10.36 mW mm$^{-2}$. The inset shows the dark current. **e** Optical switching performance of the AgAu/GaN photodetectors at 0.1 V at 10.36 mW/mm$^2$. **f-h** Summary plots of the R, D and EQE as function of photon power variation at 0.1 V

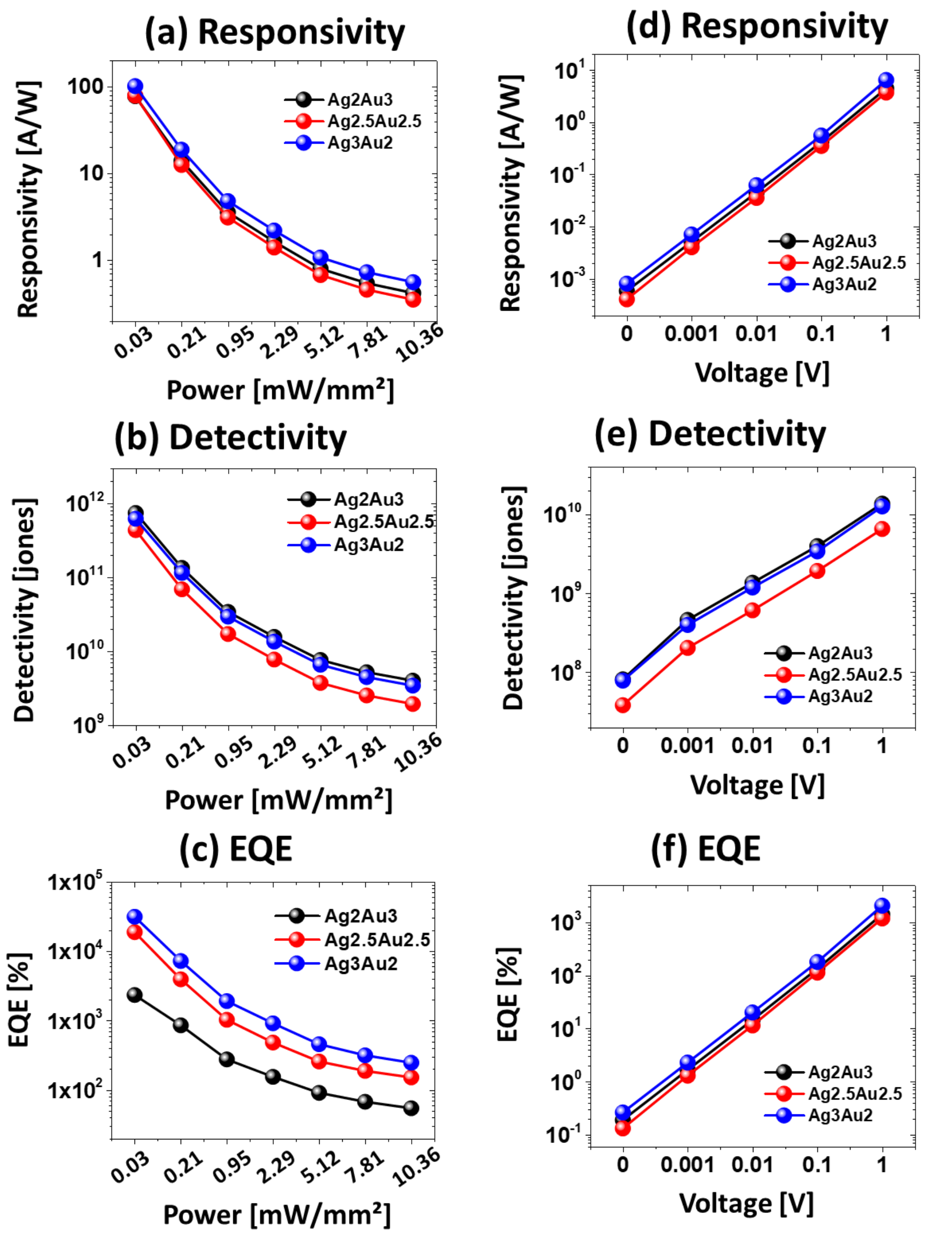


**Fig. S16 a-c** Summary plots of the responsivity, detectivity and EQE of the $Ag_3Au_2$/GaN, $Ag_{2.5}Au_{2.5}$/GaN and $Ag_2Au_3$/GaN photodetectors based on the light intensity variation at fixed 0.1 V bias. **d-f** Summary of the responsivity, detectivity and EQE based on the voltage variation at fixed illumination of 385 nm at 10.36 mW $mm^{-2}$

**Table S1** Summary of the average diameter (AD) and height (AH) of various mono-/bi-metallic NPs fabricated on GaN

| **Samples** | **AD [nm]** | **AH [nm]** | $R_q$ **[nm]** | **SAR [%]** |
|---|---|---|---|---|
| Ag/GaN | 102 | 27 | 13.23 | 13.93 |
| Au/GaN | 105 | 25 | 8.23 | 10.40 |
| Ag-1/GaN | 37.6 | 6 | 1.91 | 1.80 |
| Au-1/GaN | 32.3 | 8 | 1.89 | 1.51 |
| Ag4Au3/GaN | 114 | 26 | 11.44 | 15.86 |
| Ag3.5Au3.5/GaN | 116 | 27 | 10.95 | 14.19 |
| Ag3Au4/GaN | 118 | 30 | 11.04 | 13.67 |
| Ag3Au2/GaN | 97 | 19 | 8.58 | 10.08 |
| Ag2.5Au2.5/GaN | 100 | 22 | 7.13 | 8.69 |
| Ag2Au3/GaN | 103 | 22 | 6.67 | 7.76 |

**Table S2** Summary of the responsivity (R), detectivity (D) and external quantum efficiency (EQE) of the bare GaN, Ag, Au photodetectors as function of illumination power variation at a constant bias of 0.1 V

| **Power [mW/mm$^2$]** | **Responsivity [A/W]** | | | **Detectivity [Jones]** | | | **EQE [%]** | | |
|---|---|---|---|---|---|---|---|---|---|
| | **GaN** | **Ag** | **Au** | **GaN** | **Ag** | **Au** | **GaN** | **Ag** | **Au** |
| **0.03** | 7.26 | 58.5 | 97.4 | 2.91E+11 | 9.83E+11 | 1.94E+12 | 2339 | 18800 | 31370 |
| **0.21** | 2.66 | 12.2 | 22.5 | 1.07E+11 | 2.06E+11 | 4.48E+11 | 855 | 3940 | 7230 |
| **0.95** | 0.86 | 3.21 | 5.97 | 3.44E+10 | 5.40E+10 | 1.18E+11 | 276 | 1030 | 1911 |
| **2.29** | 0.48 | 1.5 | 2.8 | 1.93E+10 | 2.53E+10 | 5.67E+10 | 155 | 484 | 916 |
| **5.12** | 0.28 | 0.802 | 1.4 | 1.14E+10 | 1.35E+10 | 2.83E+10 | 91 | 258 | 457 |
| **7.81** | 0.20 | 0.586 | 0.9 | 8.45E+09 | 9.86E+09 | 1.96E+10 | 67 | 189 | 316 |
| **10.36** | 0.17 | 0.471 | 0.7 | 6.81E+09 | 7.92E+09 | 1.54E+10 | 54 | 152 | 247 |

**Table S3** Summary of the *R*, *D* and *EQE* of the bare GaN, Ag, Au based on the bias variation at 10.36 mW/mm$^2$

| Voltage [V] | Responsivity [A/W] | | | Detectivity [Jones] | | | EQE [%] | | |
|---|---|---|---|---|---|---|---|---|---|
| | GaN | Ag | Au | GaN | Ag | Au | GaN | Ag | Au |
| **0** | 3.16E-4 | 4.78E-4 | 0.00132 | 1.29E+08 | 1.09E+08 | 3.19E+08 | 0.102 | 0.154 | 0.425 |
| **0.001** | 0.0022 | 0.00425 | 0.00923 | 8.25E+08 | 6.45E+08 | 1.69E+09 | 0.71 | 1.37 | 2.97 |
| **0.01** | 0.0193 | 0.0384 | 0.0803 | 2.44E+09 | 1.97E+09 | 5.04E+09 | 6.22 | 12.4 | 25.9 |
| **0.1** | 0.17 | 0.471 | 0.77 | 6.81E+09 | 7.92E+09 | 1.54E+10 | 54.8 | 152 | 248 |
| **1** | 2.01 | 4.05 | 7.88 | 2.46E+10 | 2.48E+10 | 4.81E+10 | 648 | 1300 | 2540 |

**Table S4** Summary of the *R*, *D* and *EQE* of the $Ag_4Au_3$, $Ag_{3.5}Au_{3.5}$, $Ag_3Au_4$ as function of illumination power variation at a constant bias of 0.1 V

| Power [mW/mm$^2$] | Responsivity [A/W] | | | Detectivity [Jones] | | | EQE [%] | | |
|---|---|---|---|---|---|---|---|---|---|
| | $Ag_4Au_3$ | $Ag_{3.5}Au_{3.5}$ | $Ag_3Au_4$ | $Ag_4Au_3$ | $Ag_{3.5}Au_{3.5}$ | $Ag_3Au_4$ | $Ag_4Au_3$ | $Ag_{3.5}Au_{3.5}$ | $Ag_3Au_4$ |
| **0.03** | 112 | 75.8 | 48.4 | 2.40E+12 | 5.15E+11 | 2.14E+12 | 36000 | 24400 | 15600 |
| **0.21** | 24.4 | 11.3 | 8.84 | 5.25E+11 | 7.67E+10 | 3.90E+11 | 7860 | 3630 | 2850 |
| **0.95** | 6.47 | 2.75 | 2.5 | 1.39E+11 | 1.87E+10 | 1.10E+11 | 2080 | 885 | 804 |
| **2.29** | 3.07 | 1.23 | 1.22 | 6.60E+10 | 8.34E+09 | 5.37E+10 | 988 | 395 | 392 |
| **5.12** | 1.52 | 0.588 | 0.613 | 3.27E+10 | 4.00E+09 | 2.70E+10 | 489 | 189 | 197 |
| **7.81** | 1.05 | 0.398 | 0.44 | 2.25E+10 | 2.71E+09 | 1.94E+10 | 337 | 128 | 142 |
| **10.36** | 0.814 | 0.305 | 0.35 | 1.75E+10 | 2.07E+09 | 1.54E+10 | 262 | 98.1 | 113 |

**Table S5** Summary of the *R*, *D* and *EQE* of the $Ag_4Au_3$, $Ag_{3.5}Au_{3.5}$, $Ag_3Au_4$ photodetectors with the large size NPs as function of bias variation at 10.36 mW/mm$^2$

| Voltage [V] | Responsivity [A/W] | | | Detectivity [Jones] | | | EQE [%] | | |
|---|---|---|---|---|---|---|---|---|---|
| | **$Ag_4Au_3$** | **$Ag_{3.5}Au_{3.5}$** | **$Ag_3Au_4$** | **$Ag_4Au_3$** | **$Ag_{3.5}Au_{3.5}$** | **$Ag_3Au_4$** | **$Ag_4Au_3$** | **$Ag_{3.5}Au_{3.5}$** | **$Ag_3Au_4$** |
| **0** | 5.96E-4 | 3.5E-4 | 0.00114 | 3.27E+08 | 3.44E+07 | 2.23E+08 | 0.366 | 0.113 | 0.192 |
| **0.001** | 0.00435 | 0.00359 | 0.00872 | 1.83E+09 | 1.80E+08 | 1.78E+09 | 2.81 | 1.16 | 1.4 |
| **0.01** | 0.0381 | 0.0316 | 0.0771 | 5.45E+09 | 5.30E+08 | 5.28E+09 | 24.8 | 10.2 | 12.3 |
| **0.1** | 0.35 | 0.305 | 0.814 | 1.75E+10 | 2.07E+09 | 1.54E+10 | 262 | 98.1 | 113 |
| **1** | 3.8 | 3.29 | 7.69 | 4.71E+10 | 5.67E+09 | 5.11E+10 | 2480 | 1060 | 1220 |

**Table S6:** Summary of the *R*, *D* and *EQE* of the $Ag_3Au_2$, Ag2$_{.5}$Au$_{2.5}$, $Ag_2Au_3$ photodetectors with the smaller size NPs as function of illumination power variation at a constant bias of 0.1 V

| Power [mW/mm$^2$] | Responsivity [A/W] | | | Detectivity [Jones] | | | EQE [%] | | |
|---|---|---|---|---|---|---|---|---|---|
| | **$Ag_3Au_2$** | **$Ag_{2.5}Au_{2.5}$** | **$Ag_2Au_3$** | **$Ag_3Au_2$** | **$Ag_{2.5}Au_{2.5}$** | **$Ag_2Au_3$** | **$Ag_3Au_2$** | **$Ag_{2.5}Au_{2.5}$** | **$Ag_2Au_3$** |
| **0.03** | 102 | 79.3 | 78.3 | 6.22E+11 | 4.35E+11 | 7.45E+11 | 32900 | 25500 | 25219 |
| **0.21** | 18.9 | 12.6 | 14.11 | 1.15E+11 | 6.88E+10 | 1.34E+11 | 6080 | 4050 | 4543 |
| **0.95** | 4.83 | 3.12 | 3.6 | 2.94E+10 | 1.71E+10 | 3.41E+10 | 1550 | 1000 | 1154 |
| **2.29** | 2.21 | 1.41 | 1.64 | 1.35E+10 | 7.72E+09 | 1.56E+10 | 711 | 454 | 529 |
| **5.12** | 1.08 | 0.679 | 0.8 | 6.56E+09 | 3.72E+09 | 7.62E+09 | 347 | 219 | 257 |
| **7.81** | 0.731 | 0.46 | 0.55 | 4.46E+09 | 2.52E+09 | 5.20E+09 | 236 | 148 | 175 |
| **10.36** | 0.561 | 0.353 | 0.4 | 3.42E+09 | 1.93E+09 | 4.00E+09 | 181 | 114 | 135 |

**Table S7** Summary of the *R*, *D* and *EQE* of the bare $Ag_3Au_2$, $Ag2_{.5}Au_{2.5}$, $Ag_2Au_3$ photodetectors with smaller size NPs as function of bias variation at a constant power of10.36 mW/mm$^2$

| **Voltage [V]** | **Responsivity [A/W]** | | | **Detectivity [Jones]** | | | **EQE [%]** | | |
|---|---|---|---|---|---|---|---|---|---|
| | **$Ag_3Au_2$** | **$Ag_{2.5}Au_{2.5}$** | **$Ag_2Au_3$** | **$Ag_3Au_2$** | **$Ag_{2.5}Au_{2.5}$** | **$Ag_2Au_3$** | **$Ag_3Au_2$** | **$Ag_{2.5}Au_{2.5}$** | **$Ag_2Au_3$** |
| **0** | 8.12E-4 | 4.06E-4 | 5.87E-4 | 7.85E+07 | 3.83E+07 | 8.15E+07 | 0.262 | 0.131 | 0.189 |
| **0.001** | 0.00711 | 0.00404 | 0.00517 | 3.99E+08 | 2.04E+08 | 4.62E+08 | 2.29 | 1.3 | 1.67 |
| **0.01** | 0.0627 | 0.0356 | 0.0454 | 1.19E+09 | 6.11E+08 | 1.37E+09 | 20.2 | 11.5 | 14.6 |
| **0.1** | 0.561 | 0.353 | 0.421 | 3.42E+09 | 1.93E+09 | 4.00E+09 | 181 | 114 | 135 |
| **1** | 6.5 | 3.73 | 4.48 | 1.27E+10 | 6.58E+09 | 1.38E+10 | 2090 | 1200 | 1440 |
